# Autonomous Microrobots for Spatiotemporally Active Therapeutic Delivery and Controlled Release

**DOI:** 10.34133/cbsystems.0617

**Published:** 2026-06-29

**Authors:** Shihao Zhong, Anping Wu, Shanming Bai, Henwei Huang, Toshio Fukuda, Huaping Wang

**Affiliations:** ^1^ Intelligent Robotics Institute, School of Mechatronical Engineering, Beijing Institute of Technology, Beijing 100081, China.; ^2^ Key Laboratory of Biomimetic Robots and Systems (Beijing Institute of Technology), Ministry of Education, Beijing 100081, China.; ^3^ School of Medical Technology, Beijing Institute of Technology, Beijing 100081, China.; ^4^ School of Electrical and Electronic Engineering, Nanyang Technological University, Singapore 639798, Singapore.; ^5^ Lee Kong Chian School of Medicine, Nanyang Technological University, Singapore 639798, Singapore.; ^6^ Department of Micronano Systems Engineering, Nagoya University, Nagoya 464-8603, Japan.

## Abstract

The clinical efficacy of many conventional passive drug delivery systems is frequently constrained by their low targeting efficiency, important off-target toxicity, and inadequate capacity for traversing biological barriers. Autonomous microrobots, as miniature intelligent platforms capable of active navigation and on-demand responsiveness, offer an active delivery strategy for achieving spatiotemporally precise targeted therapy. This review aims to systematically consolidate and critique the theoretical foundations, key technologies, cutting-edge applications, and future challenges of this emergent interdisciplinary field. We first provide an in-depth analysis of the technological frameworks underpinning the 2 core functionalities: targeted delivery and on-demand release. This encompasses a diverse array of propulsion and navigation strategies—from chemical and physical fields to biohybrid systems—as well as programmed drug release mechanisms responsive to endogenous and exogenous stimuli. Building on this, we introduce a hierarchical paradigm organized by biological-barrier traversal capability to review the preclinical progress of microrobots, from localized delivery in accessible body cavities to deep-tissue and trans-barrier applications. This function-oriented framework more directly links microrobot design to the progressive physiological constraints encountered in vivo, thereby providing a more integrated and translationally relevant perspective on biomedical applications and clinical potential. Concurrently, this paper examines the bottlenecks impeding their clinical translation, including biosafety, systemic controllability, and regulatory science. Looking forward, the deep integration of microrobotics with smart materials, artificial intelligence, and theranostic systems is poised to cultivate a new generation of intelligent medical robots capable of personalized treatment via closed-loop manners.

## Introduction

Pharmacotherapy is the cornerstone of modern medicine in combating diseases, injuries, and functional disorders [1]. Traditional drug administration routes, such as oral or intravenous injection, while widely used, present challenges due to systemic distribution [2]. These methods distribute drug molecules throughout the entire circulatory system, causing them not only to act on the target lesion but also to affect healthy tissues and organs, thereby inducing significant off-target toxicity and adverse reactions. Achieving effective local therapeutic concentrations often requires higher dosages, which exacerbates systemic side effects and reduces patient compliance. To overcome this dilemma, passive-targeting drug delivery systems like nanomedicine have emerged [3,4]. These systems leverage the size effects of nanocarriers and the enhanced permeability and retention effect in tumors to improve drug accumulation at the lesion site. However, their efficiency remains limited [5]. As “passive drifting” carriers, they rely on random circulation and slow diffusion, resulting in typically less than 1% of the administered dose reaching a solid tumor [6]. Importantly, they lack active navigation capabilities, making it difficult to effectively cross physiological barriers [7] such as the blood–brain barrier, the dense extracellular matrix (ECM) of tumors, or mucus layers. They are also unable to move against the bloodstream to reach deep into poorly perfused areas of a lesion [8]. Therefore, developing next-generation drug delivery platforms with active navigation, precise targeting, and controllable release is a critical challenge in modern pharmaceutics and biomedical engineering [9].

An ideal drug delivery strategy should achieve “spatiotemporal precision” [10,11]. In the spatial dimension, this means “targeted delivery”, which is the ability to transport the therapeutic payload efficiently and specifically to specific pathological tissues, cells, or even subcellular organelles. This achieves an extremely high local drug concentration at the target site, maximizing the therapeutic effect while minimizing interference with healthy tissues. In the temporal dimension, it requires “on-demand release”, meaning that the drug release behavior can be precisely controlled according to a preset program or in response to specific endogenous or exogenous signals. This controllable release not only maintains a stable drug concentration within the therapeutic window, avoiding the toxic peaks of “burst release” and subsequent insufficient concentrations, but also can be synchronized with the physiological rhythms of the disease or triggered by the appearance of disease biomarkers, enabling intelligent and personalized therapeutic intervention. Spatiotemporally precise drug delivery is poised to not only revolutionize the treatment paradigms for major challenging diseases such as cancer and neurodegenerative disorders but also to significantly enhance the safety and quality of life for patients by reducing the total dosage and administration frequency, holding profound clinical significance [12,13].

**Fig. 1.**
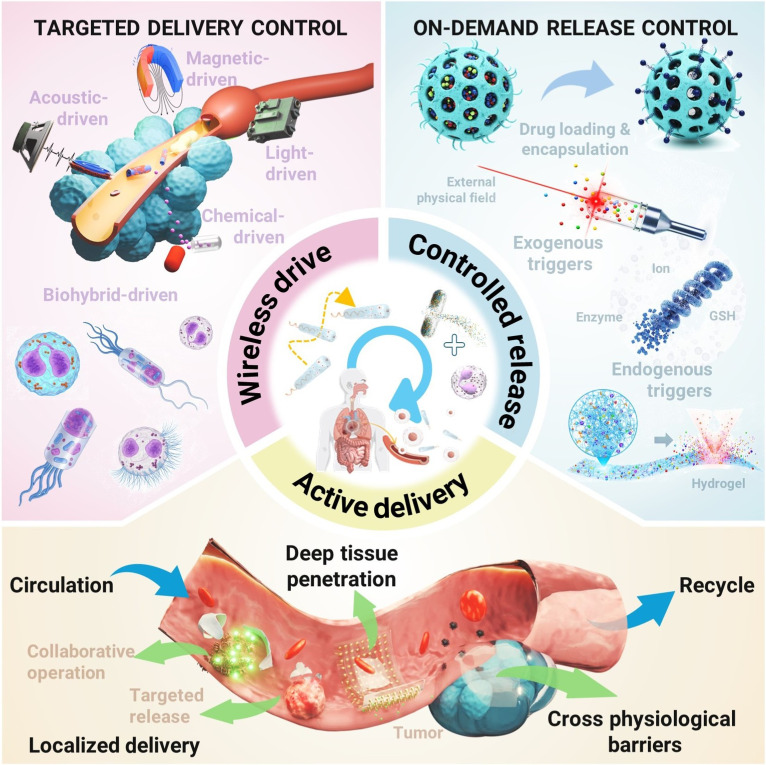
Overview of the autonomous micro/nanorobots system for spatiotemporally active therapeutic delivery and controlled release.

Achieving spatiotemporally precise delivery requires that carriers transition from “passive” to “active”. In recent years, the convergence of materials science, micro/nanofabrication, and robotics has led to the emergence of autonomous robots as a revolutionary drug delivery platform with immense potential [14,15]. These micro- or nanoscale machines efficiently convert chemical or external physical energy into mechanical motion, achieving active propulsion in complex physiological environments [16–18]. Furthermore, the motion of these robots can be precisely controlled by external energy fields [19,20], or they can utilize their biohybrid designs to achieve autonomous chemotactic navigation toward specific physiological signals [21]. This significant active locomotion capability enables them to overcome physiological barriers, move against fluid flow, and penetrate deep into tissues that are inaccessible to traditional drugs [22,23]. Additionally, by integrating smart responsive materials into their structure [24], robots can achieve “on-demand” controllable release of their payload in response to specific endogenous or exogenous trigger signals upon reaching the intended target. By integrating functions such as “sensing”, “actuation”, “navigation”, and “execution”, robots can provide an unparalleled technological platform for advancing drug delivery from the era of passive targeting to an era of active, intelligent, and precise therapy (Fig. 1).

This paper systematically reviews the theoretical foundations, key technologies, innovative applications, and future challenges of autonomous robots in precise drug delivery, a field with revolutionary potential. Starting from the core functional requirements for achieving spatiotemporally precise drug delivery, the paper will deeply deconstruct the 2 major technology systems of robots: targeted delivery control and on-demand release control. The former will systematically elaborate on the diverse active propulsion mechanisms, from chemical and physical fields to biohybrid systems, and will further analyze the exogenous navigation and autonomous targeting strategies for achieving precise arrival. The latter will comprehensively review the technological pathways from efficient drug encapsulation to intelligent release in response to endogenous/exogenous signals, and even programmed synergistic delivery. Building on this, the present review further focuses on the preclinical applications of microrobots. Compared with traditional review frameworks, it introduces a hierarchical paradigm based on biological-barrier traversal capability, organizing relevant studies according to the increasing complexity of barriers encountered during in vivo delivery, from localized delivery within body cavities to systemic circulation, deep tissue penetration, and targeting of immune-privileged sites. This framework provides a clearer view of the current application landscape, developmental trajectory, and potential of this technology. However, despite the promising prospects, translating this technology from the laboratory to the clinic still faces severe multidimensional challenges, including biosafety, system robustness, and ethical and regulatory issues, which will be analyzed in this paper. Finally, based on the current technological bottlenecks, this paper will look forward to the future development trends in this field, such as new materials, new intelligence, new functions, and recent technology integration. Through this multilevel and cross-dimensional systematic review, we hope to provide a comprehensive technical blueprint and forward-looking strategic thinking for researchers, clinicians, and industry professionals engaged in related research, and jointly promote the eventual clinical translation of this disruptive medical technology.

## Targeted Delivery Control

To achieve precise targeted delivery of robots in complex biological environments, the primary issue to be addressed is their “locomotion”, that is, how to drive and navigate them. The propulsion mechanisms of robots can be categorized into 2 main types: passive transport and active propulsion. In certain specific application scenarios, robots can utilize natural physiological forces for passive transport, such as moving along with the flow of biological fluids like blood and lymph or through the peristalsis of the gastrointestinal tract [25–27]. Although simple, this method’s trajectory and targeting efficiency are difficult to control, hindering precise delivery. Active and controllable propulsion is the core feature of autonomous robots. It converts external energy into mechanical motion through various mechanisms and, based on closed-loop control algorithms, overcomes external disturbances to achieve directed movement.

### Active promotion mechanism

**Fig. 2.**
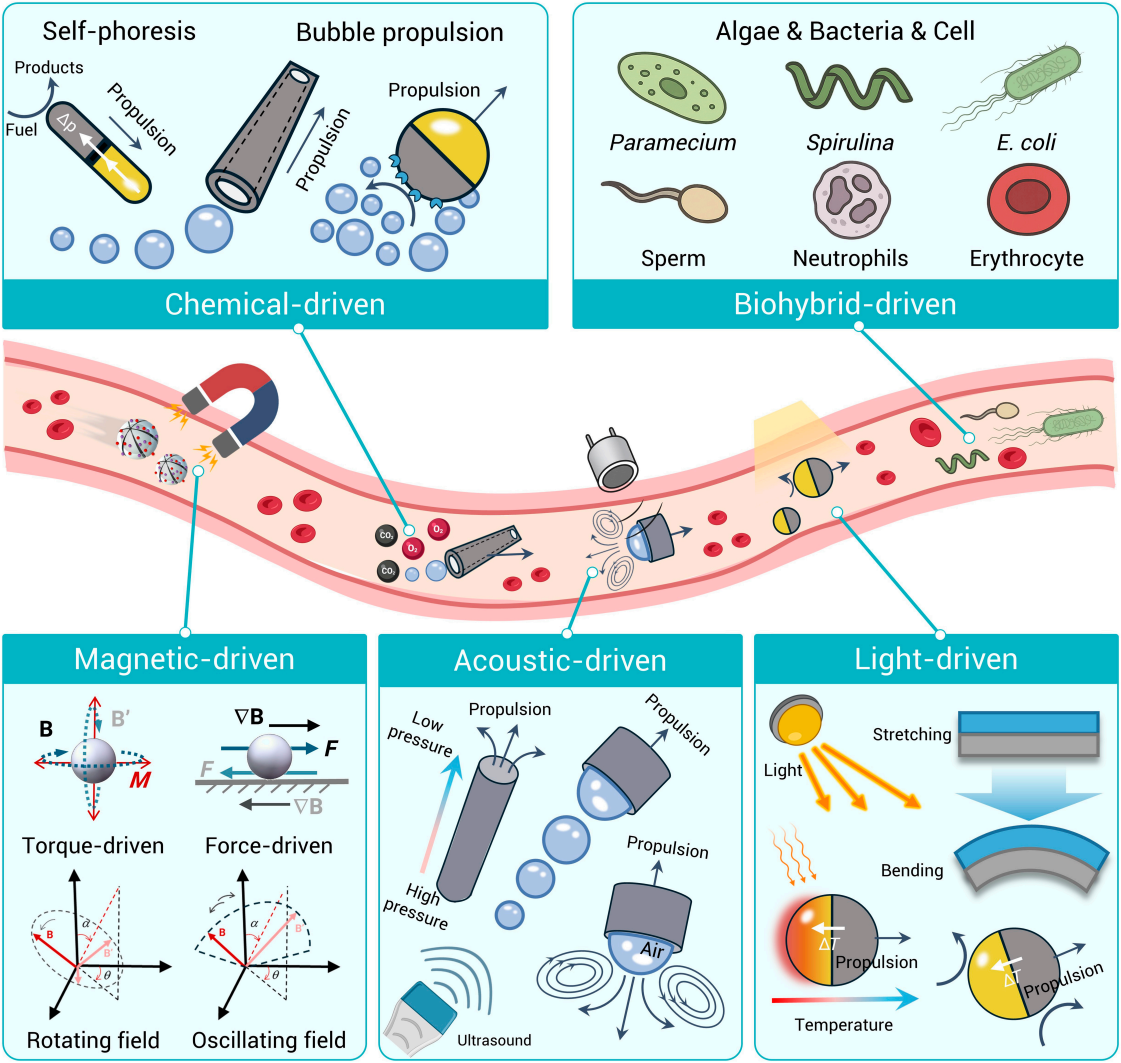
Propulsion and steering mechanisms of micro/nanorobots.

The active propulsion mechanism of a robot is the core technology that enables it to convert energy from its surrounding environment or from onboard sources into kinetic energy for directed motion. This mechanism allows the robot to overcome the dominant viscous forces, enabling advanced functions such as autonomous navigation, upstream motility against fluid flow, and penetration of biological barriers (Fig. 2).

### Chemical propulsion

Chemically propelled robots utilize substances in their local environment as fuel, achieving propulsion and behavioral control by generating chemical gradients or bubble jets through asymmetric reactions [16].

A classic approach involves using inorganic catalysts, particularly noble metals like platinum (Pt), to decompose hydrogen peroxide (H_2_O_2_). Pioneering early studies demonstrated the autonomous movement of bimetallic nanowires composed of different metal segments (e.g., Pt-Au) in an H_2_O_2_ solution through a “self-electrophoresis” mechanism [28,29]. In this process, H_2_O_2_ undergoes oxidation and reduction reactions on the different metal segments of the nanowire, creating a proton gradient and a built-in electric field that drives the nanowire’s movement. Another classic inorganic catalytic design involves tubular or Janus-structured robots. The inner wall of tubular robots is typically coated with a catalytic layer such as Pt. H_2_O_2_ enters the tube and decomposes, producing oxygen bubbles that are ejected from one end, creating a recoil force that propels the robot forward [30]. Janus robots, on the other hand, usually have 1 hemisphere modified with a Pt catalytic cap. The asymmetric catalytic reaction generates a chemical gradient, driving their movement via self-diffusiophoresis [31]. However, when evaluating in vivo feasibility, chemically powered systems relying on exogenous H_2_O_2_ fuels face substantial barriers to broad clinical application. Because even low concentrations of H₂O₂ can induce oxidative stress and cellular toxicity, the application of these foundational inorganic systems is largely restricted to in vitro demonstrations or highly localized, well-controlled experimental settings.

Another common method utilizes reactive metals [32] that react with water or physiological fluids (e.g., stomach acid) to gain thrust by ejecting bubbles. For instance, magnesium (Mg)-based robots produce hydrogen (H₂) bubbles by reacting with water for propulsion [33,34], while zinc (Zn)-based robots achieve locomotion in acidic environments like the stomach through a similar H₂-generating reaction [35].

To address these biocompatibility challenges and advance toward clinical translation, enzymes are employed as catalysts [36]. Common enzymes like urease and catalase [37] utilize endogenous fuels such as urea under mild physiological conditions. For instance, urease-powered robots move via ion self-diffusiophoresis by decomposing urea to create an ion gradient [38,39]. Similarly, catalase can replace noble metal catalysts to achieve propulsion at lower, more biocompatible H₂O₂ concentrations [40]. Nevertheless, to minimize safety risks, their in vivo applications are predominantly explored within specific pathological microenvironments (such as tumors) where endogenous H₂O₂ naturally accumulates, thereby reducing the reliance on introducing external fuels.

#### Physical-field-driven propulsion

Physical field-driven propulsion is currently the most mature and safest method for in vivo applications. It primarily utilizes external fields (such as magnetic, acoustic, or light fields) to provide wireless energy and achieve programmable propulsion [23,24].

Magnetic Fields: It is a clinically promising method for in vivo propulsion due to their deep tissue penetration, minimal damage, and high safety [17,41,42]. Magnetic propulsion is mainly achieved through 2 mechanisms: first, using a magnetic field gradient to generate a magnetic force that directly pulls and navigates the robot [43]; second, using a time-varying rotating or oscillating magnetic field to generate a magnetic torque. This torque drives robots with specific geometries (e.g., helical or flexible chain-like structures) to generate propulsive force through rotation or oscillation, mimicking the “corkscrew-like” motion or flagellar swimming of microorganisms [44–46]. By programmatically controlling the direction, strength, and frequency of the external magnetic field, precise regulation of the robot’s direction and speed can be achieved [47].

Acoustic Fields: Typically, in the form of ultrasound, acoustic fields are another form of energy with good penetration through biological tissues [48]. When an acoustic field interacts with robots that have an asymmetric geometry or density distribution, it creates an unbalanced acoustic streaming in their vicinity, thus generating a net thrust to propel the robot [49,50]. Furthermore, precise control of microbubble oscillations using an acoustic field can also produce powerful propulsion. A common design involves stably trapping single or multiple microbubbles within the robot’s cavity structure [51,52]. When excited by ultrasound at a specific frequency, these bubbles undergo resonant oscillations, generating intense local acoustic streaming vortices around them that provide thrust to the robot. A more powerful method involves using ultrasound to trigger the rapid vaporization of a propellant (such as a perfluorocarbon liquid) encapsulated inside the robot, producing a high-speed jet of gas bubbles [53].

Light Fields: Light-driven propulsion offers the advantage of high spatiotemporal resolution, but its penetration depth in biological tissues is limited. Therefore, it is mainly suitable for applications on the body surface or in superficial tissues (such as the eye) [54,55]. Light-driven propulsion mechanisms are primarily divided into 3 categories: first, photochemical propulsion, which uses light energy as a catalyst or trigger to initiate or accelerate chemical reactions on the robot’s surface, generating a chemical gradient or bubbles for propulsion [56]. Second, photothermal propulsion, which utilizes the photothermal effect of materials (like gold nanoparticles) to convert light energy into a local thermal gradient, driving the robot’s movement through thermophoresis [57,58]. Third, photomechanical propulsion, which uses light to directly induce reversible physical deformations (such as bending or shrinking) in photosensitive polymer materials, thereby generating mechanical motion [59].

#### Biological hybrid propulsion

Biohybrid propulsion is a revolutionary strategy that utilizes living biological entities (such as bacteria, cells, or microalgae) as “engines”, cleverly harnessing their innate motility, environmental sensing capabilities, and biocompatibility to drive functional carriers for completing complex medical tasks [18,60].

Bacteria: Flagellated bacteria have become the most extensively studied biological “engines” due to their powerful motility and chemotaxis. Magnetotactic bacteria, such as *Magnetospirillum magneticum* AMB-1, contain magnetosome chains that act as a built-in “compass”, enabling efficient targeted drug delivery under the guidance of an external magnetic field [61,62]. For nonmagnetic but highly motile bacteria, such as *Escherichia coli* or *Salmonella*, researchers typically couple them with artificial magnetic components (e.g., magnetic nanoparticles or microrods). This strategy confers the ability for magnetic navigation via an external field while preserving the bacteria’s innate chemotactic capabilities [63,64].

Microalgae: *Spirulina* has emerged as a highly promising biological motor due to its unique natural helical structure. After being coated with magnetic nanoparticles (e.g., Fe_3_O_4_), this helical shape enables efficient “corkscrew-like” propulsion in a rotating magnetic field [65,66]. Additionally, *Spirulina* possesses intrinsic autofluorescence, facilitating in vivo imaging and tracking, while its good biocompatibility and biodegradability also add to its advantages for biomedical applications [67,68].

Cells: Employing a mammal’s own cells as propulsion units or carriers is a highly promising strategy in biohybrid robotics, as it maximizes biocompatibility and reduces the risk of immune rejection. (a) Sperm cells: Functioning as efficient biological motors via powerful flagellar propulsion, sperm cells are used to construct “spermbots”. These are created by trapping a single sperm cell in a magnetic microtube, where the sperm provides forward thrust and the microtube enables navigation by an external magnetic field [69,70]; (b) Immune cells: Immune cells, such as neutrophils, possess natural chemotaxis, allowing them to autonomously migrate toward tumor or inflammation sites and cross physiological barriers like the blood–brain barrier. By inducing neutrophils to engulf drug-loaded magnetic nanogels, “neutrobots” are formed. These retain the cell’s innate ability to cross the blood–brain barrier while gaining magnetic controllability, offering new therapeutic avenues for diseases like brain tumors [71,72]; (c) Red blood cells (RBCs): Although lacking active motility, RBCs are ideal drug carriers due to their excellent biocompatibility, deformability, and long circulation lifespan. “RBC-bots” are constructed by attaching drug-loaded RBCs to bacterial motors or by encapsulating magnetic nanoparticles within them. This imparts active movement and targeted navigation capabilities while preserving their natural immune-evasive properties [73].

### Navigation and targeting strategies

**Fig. 3.**
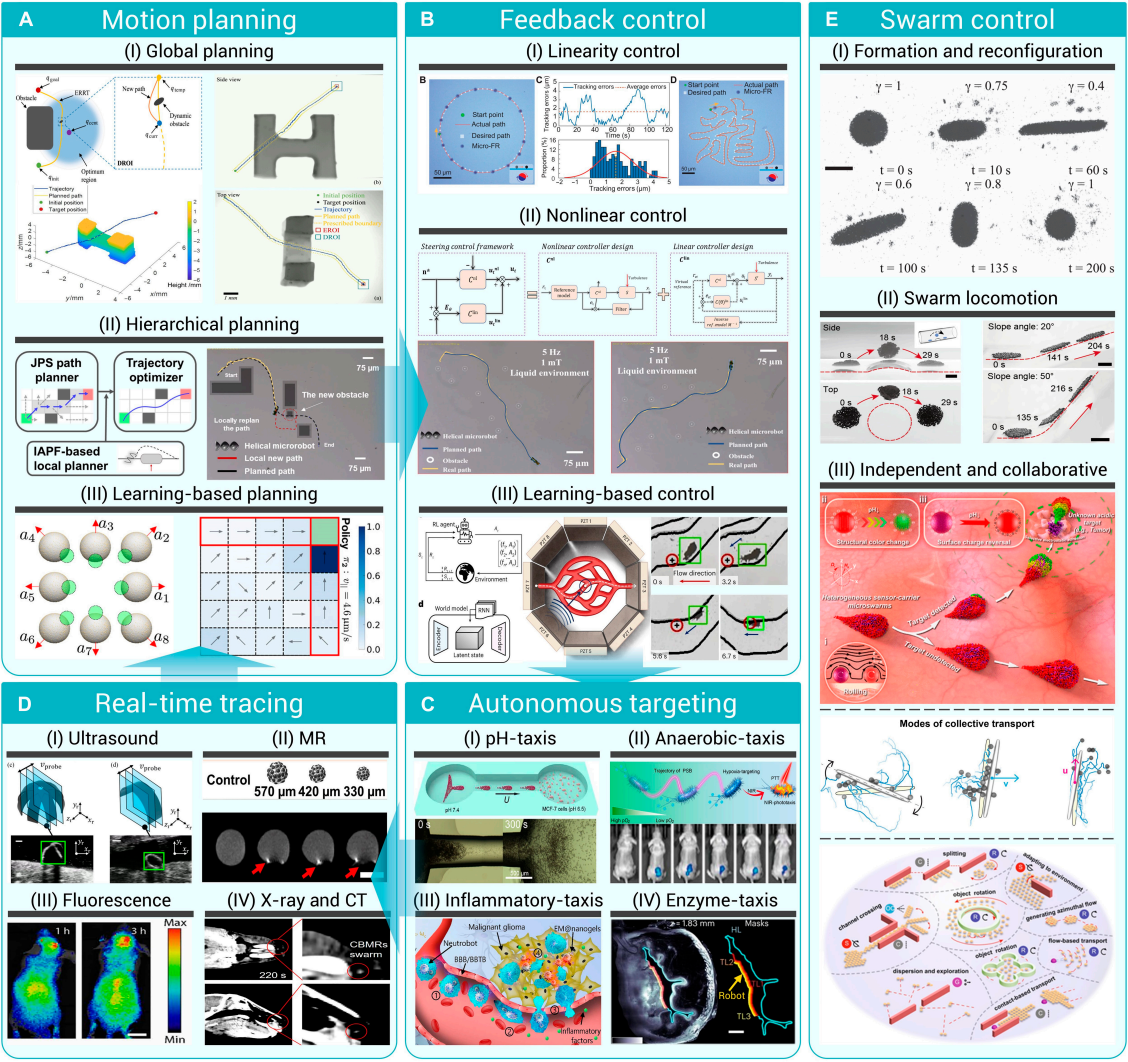
Motion navigation control. (A) Motion planning algorithms. (I) The global path planned by the enhanced rapidly exploring random tree algorithm. Scale bar: 1 mm. Reproduced or adapted with permission from Ref. [92], Copyright 2022 IEEE. (II) Global planning and local emergency replanning. Scale bar: 75 μm. Reproduced or adapted with permission from Ref. [44], Copyright 2024 IEEE. (III) Reinforcement learning-based motion planning. Reproduced or adapted with permission from Ref. [101], Copyright 2021 AAAS. (B) Motion feedback control. (I) Linear proportional-integral-derivative (PID) control. Scale bar: 50 μm. Reproduced or adapted with permission from Ref. [102], (CC BY-NC 4.0). (II) Compound nonlinear control. Scale bar: 75 μm. Reproduced or adapted with permission from Ref. [105], Copyright 2024 IEEE. (III) Reinforcement learning control. Scale bar: 200 μm. Reproduced or adapted with permission from Ref. [111], (CC BY 4.0). (C) Autonomous targeting motion. (I) Microrobots migrate in response to local pH gradients. Scale bar: 500 μm. Reproduced or adapted with permission from Ref. [114], (CC BY 4.0). (II) Anaerobic bacteria selectively target hypoxic regions. Reproduced or adapted with permission from Ref. [116], Copyright 2020 ACS. (III) Microrobots move along the concentration gradient of inflammatory cytokines. Reproduced or adapted with permission from Ref. [71], Copyright 2021 AAAS. (IV) Nanorobots aggregate in response to urease concentration gradients. Scale bar: 500 μm. Reproduced or adapted with permission from Ref. [118], (CC BY 4.0). (D) Visual feedback. (I) Ultrasound imaging. Scale bar: 1 mm. Reproduced or adapted with permission from Ref. [77], (CC BY 4.0). (II) Magnetic resonance (MR) imaging. Scale bar: 5 mm. Reproduced or adapted with permission from Ref. [86], (CC BY-NC 4.0). (III) Fluorescence imaging. Scale bar: 10 mm. Reproduced or adapted with permission from Ref. [71], Copyright 2021 AAAS. (IV) Computed tomography (CT) imaging. Scale bar: 2 cm. Reproduced or adapted with permission from Ref. [89], Copyright 2025 AAAS. (E) Swarm control. (I) Various swarm formations. Scale bar: 500 μm. Reproduced or adapted with permission from Ref. [136], Copyright 2022 IEEE. (II) Directional movement of a swarm. Scale bar: 5 mm. Reproduced or adapted with permission from Ref. [138], (CC BY-NC 4.0). (III) Independent and collaborative tasks performed by multiple swarms. Reproduced or adapted with permission from Ref. [140], Copyright 2024 ACS; [133], Copyright 2024 AAAS; [141], (CC BY-NC 4.0). BBB, blood–brain barrier.

Once capable of active propulsion, accurately steering the robots to their destination is key for targeted therapeutic delivery (Fig. 3 and Table 1).

**Table 1.** Comparison of targeted delivery capabilities of microrobots

Navigation paradigm	Propulsion Mechanism	Information source and control method	Core advantages	Key challenges and limitations
External navigation	• Physical fields • Biohybrid	• Medical imaging • Closed-loop control	• High precision and controllability • Deep tissue penetration • Human–machine interaction	• Complex clinical device integration • Limited imaging resolution/contrast • Highly environmental disturbance
Autonomous targeting	• Chemical • Biohybrid	• Pathological signals • Target cell surface • Autonomous tendency	• Intelligent and automated • High targeting specificity • Penetration into deep avascular/hypoxic regions	• Biological heterogeneity of targets • Low trajectory predictability • Susceptibility to fluidic disturbances
Collective navigation	• Mainly physical fields	• Medical imaging • Global field control • Inter-robot interactions	• Emergent functions • Swarm robustness and adaptability • Collaborative multitasking	• Highly coupled dynamics • Highly underactuated system • Swarm stability and consistency

#### Exogenous navigation paradigm

This is an “outside-in” control paradigm, where an external device generates a physical field that penetrates biological tissue for wireless, controllable, real-time manipulation of the robot [20,74]. The implementation of this paradigm relies on a complete closed-loop control system, which primarily consists of 2 core components: real-time tracking and state estimation, and trajectory planning and feedback control.

##### Real-time tracking and state estimation

Precise navigation requires real-time, accurate in vivo tracking of the robot’s position, orientation, and motion state. To this end, a variety of clinical and preclinical medical imaging modalities have been employed for this purpose [75].

Ultrasound Imaging: As a noninvasive, real-time, and portable technology, ultrasound shows great potential for tracking robots [76]. B-mode ultrasound can be used to detect the position of micron-sized magnetic robots [77]. For robots in motion, Doppler mode can acquire velocity information by detecting the frequency shift of sound waves [78] and has been used to track magnetic swarms in blood vessels [79]. Recently, ultrasound acoustic phase analysis based on raw radio frequency signals has demonstrated higher sensitivity and spatial resolution, enabling the tracking of smaller robotic movements [80]. Furthermore, integrating ultrasound contrast agents (such as microbubbles) can significantly enhance the robot’s echo signal, thereby improving imaging contrast [81].

Magnetic Resonance Imaging (MRI): MRI provides high-contrast, high-spatial-resolution, 3-dimensional (3D) imaging of soft tissues without ionizing radiation. It functions by detecting the relaxation signals of hydrogen protons within a strong magnetic field [82]. To be visualized in MRI, robots must contain materials that significantly alter the local magnetic field, such as superparamagnetic iron oxide nanoparticles, which produce distinct negative signals (signal voids) on T2-weighted images [83,84]. A unique advantage of MRI is that its powerful magnetic field can be simultaneously utilized for both the actuation and imaging of the robots, realizing a unified system for both functions [85]. For example, MRI has been used to provide real-time visualization and guidance for the delivery process of robots in a chemoembolization therapy model in a porcine liver [86].

Fluorescence Imaging: This modality operates by exciting fluorophores carried by the robots and detecting their emitted fluorescence, offering very high sensitivity and specificity [87]. It is commonly used for imaging superficial tissues in small animals, for instance, in tracking robots based on autofluorescent *Spirulina* or labeled cell-based robots [67,68].

X-ray Imaging and Computed Tomography (X-ray and CT): X-ray imaging utilizes the differential absorption of x-rays by various tissues to generate images, featuring excellent penetration depth and spatial resolution. To be detected by x-ray, robots must be composed of materials with a high atomic number (e.g., gold and titanium) or carry x-ray contrast agents [88]. Real-time x-ray fluoroscopy has been successfully applied to track the manipulation of robots in dynamic environments, such as blood vessels [89,90].

However, integrating these tracking modalities with control systems faces notable biomedical and hardware constraints. Clinically available imaging often features limited spatial resolution, making the tracking of individual microrobots highly challenging. Furthermore, inherent signal latency and relatively low frame rates in modalities like MRI can compromise the real-time responsiveness of closed-loop controls. Spatially integrating clinical imaging hardware with external actuation devices (e.g., electromagnetic coil setups) also presents a significant engineering challenge for deployment.

##### Trajectory planning and feedback control

The in vivo environment’s complex, narrow, and dynamic structures, like the dense vascular network, present enormous navigational challenges for robots. Therefore, efficient and safe path-planning algorithms are critical for achieving autonomous navigation. Researchers have successfully adapted mature algorithms from the field of macroscopic robotics to the microscale. For static or slowly changing environments, randomized or search-based planning algorithms are widely used [19,91–93]. These algorithms can efficiently find a collision-free, feasible path within complex 3D vascular networks reconstructed from medical images [94–96]. To address the dynamic nature of the in vivo environment (such as changes in blood flow and tissue peristalsis), researchers have further developed hybrid or hierarchical strategies that combine global planning with local dynamic obstacle avoidance. Such methods typically first plan a global path using a global search algorithm. Then, during the robot’s execution, a local planner, such as the artificial potential field method or the dynamic window approach, dynamically adjusts the local trajectory based on real-time sensory information to avoid dynamic obstacles in real time [44,97,98]. This hierarchical planning framework, which combines the optimality of a global path with the rapid response of local reactions, significantly enhances the navigational robustness of robots in realistic biological environments. When dealing with unstructured in vivo environments with high uncertainty, intelligent planning algorithms centered on reinforcement learning (RL) are becoming a new research frontier [99–101]. Unlike traditional methods that rely on precise environmental models, RL algorithms enable robots to autonomously learn a navigation policy through direct interaction with the environment via a “trial-and-error” mechanism. This end-to-end learning paradigm can directly map raw image sensor data to control commands, endowing the robot with intelligent decision-making and navigation capabilities.

After trajectory planning, a robust controller is needed to correct real-time deviations and ensure the robot accurately follows the path. The conventional proportional-integral-derivative controller is widely used due to its simplicity and effectiveness [102,103] and has been employed to navigate stem cell delivery robots in nude mice [104]. However, due to the strong nonlinearities, uncertainties, and physiological noise (such as pulsatile blood flow and continuous tissue deformation) present in the in vivo environment, traditional linear controllers (such as proportional-integral-derivative) often struggle to achieve desired control performance [105,106], which may lead to tracking errors that exceed acceptable clinical margins. A clinically viable closed-loop system typically requires careful management of error margins to enhance patient safety and minimize the risk of off-target tissue damage. Consequently, robust nonlinear control and intelligent control methods have become current research hotspots. Nonlinear control methods, such as sliding mode control [107] and the super-twisting algorithm [108], combined with high-gain observers [109] to estimate and compensate for unknown disturbances, can improve system robustness to some extent but typically rely on relatively accurate system dynamics models. To overcome the dependence on precise models, machine learning, particularly RL, offers a revolutionary approach to the autonomous control of robots. RL allows the robot to autonomously learn an optimal control policy through interaction and trial and error with its environment [110]. Deep RL, which combines RL with deep neural networks, can handle more complex environmental [100] and state information and has been used to control ultrasound-driven robotic swarms to avoid obstacles [111,112]. These learning-based intelligent control methods reduce the reliance on precise physical models, endowing robots with unprecedented environmental adaptability and autonomous decision-making capabilities. Nevertheless, translating RL from simulated environments to clinical reality faces substantial challenges. The current scarcity of high-fidelity in vivo data can constrain effective training, while the “black-box” nature of neural networks complicates the rigorous safety validation typically required for medical devices. Additionally, the computational load may pose challenges for real-time deployment on standard clinical hardware. While they represent a promising future direction for achieving fully autonomous navigation in complex, dynamic, and unpredictable in vivo environments, these practical bottlenecks need to be systematically addressed prior to widespread clinical adoption.

#### Autonomous targeting paradigm

The autonomous targeting paradigm endows robots with an “inside-out” form of physical intelligence, enabling them to autonomously sense specific biochemical signals in the local microenvironment and make motional decisions accordingly. This allows for chemotactic-like movement toward a target region and precise docking.

##### Chemotactic targeting

Taxis refers to the directed movement of an organism, such as a bacterium or an immune cell, in response to a chemical or physical signal gradient in the environment—a fundamental manifestation of biological intelligence. By integrating specific sensing and response elements onto robots, these biological taxis can be mimicked, enabling them to autonomously “sniff out” and track the unique chemical “scents” released by pathological tissues, thereby achieving intervention-free targeted delivery [87]. The tumor microenvironment (TME), owing to its abnormal metabolic activity, provides a natural source of signals for this taxis-based targeting.

pH-taxis: A prominent feature of the TME is its acidity (pH typically ranging from 6.5 to 6.8), which primarily results from the Warburg effect in cancer cells and the accumulation of lactic acid [113]. Building on this, researchers have enabled robots to migrate toward and locate tumors by integrating acid-sensitive or lactate-responsive modules, allowing them to move in response to low pH or low lactate concentration gradients [114]. These systems exploit the elevated lactate environment to facilitate tumor localization, offering a promising strategy for enhanced targeting and precision therapy.

Oxygen-taxis: The core region of solid tumors is often in a state of hypoxia [115]. Capitalizing on this characteristic, researchers have engineered biohybrid robots from naturally aerotactic organisms, such as magnetotactic bacteria. Under the initial guidance of an external magnetic field, these robots can leverage their intrinsic aerotaxis to autonomously penetrate deep into the hypoxic core of tumors, precisely delivering drugs to regions that are difficult for conventional therapies to reach [116].

Enzyme–substrate taxis: Beyond exploiting natural gradients in the pathological environment, a strategy involves modifying the surface of robots with specific enzymes, enabling them to move toward the concentration gradient of their substrate molecules. This approach has shown great potential in specific disease models [117]. For instance, urease-powered robots can move toward urea-rich environments, which not only provides fuel for their propulsion but also serves as a targeting mechanism [118].

Inflammation-taxis: Inflammation is a common feature of many diseases, including cancer. Inflammatory sites release many chemokines (e.g., interleukin-8, tumor necrosis factor-α, and monocyte chemoattractant protein-1) that can be utilized by robots [119]. By modifying the robot surface with receptors (such as chemokine receptor proteins) or integrating biomimetic sensing modules, the robots can mimic the chemotactic behavior of immune cells, thereby spontaneously migrating toward the site of inflammation. For example, robots camouflaged with neutrophil membranes not only retain the natural responsiveness to inflammatory factors but also have an enhanced ability to cross the vascular endothelium and accumulate in inflamed tissues [120]. More advanced designs have incorporated real-time sensing modules, allowing the robots to integrate multiple signals and adjust their course in complex biofluidic environments, thus improving targeting accuracy [71].

By decorating robot surfaces with targeting ligands (e.g., antibodies and aptamers), they recognize and bind receptors overexpressed on target cells/tissues, providing precise anchoring at the destination. Monoclonal antibodies or fragments enable high-affinity recognition of tumor antigens [121]; in ADC-like designs, they also drive internalization, and anti-HER2-modified, magnetically driven robots show selective adhesion and enhanced local delivery in breast cancer models [122]. SELEX-derived nucleic acid ligands offer compact, low-immunogenic targeting [123]; cMet-aptamer constructs bind and are endocytosed by high-cMet tumor cells and can couple targeting with light-triggered release [124]. In addition, small molecules such as folic acid exploit differential folate-receptor expression to achieve selective tumor targeting [125].

Despite their strong promise, chemotaxis-inspired targeting strategies are still at relatively early stages of experimental validation. For example, FOF1-adenosine triphosphatase-embedded chromatophore nanorobots have shown proton chemotaxis and tumor targeting in both in vitro and in vivo settings [126], while magnetic torque-driven living microrobots based on AMB-1 were first validated in 3D tumor spheroids and then further evaluated in murine tumor models [62]. However, large-animal validation of autonomous chemotactic targeting remains scarce. In addition, endogenous cues such as acidity, hypoxia, enzyme enrichment, and inflammatory signaling are not absolutely tumor-specific and may also be present in other pathological tissues, creating a risk of false-positive targeting [127]. This challenge is further amplified by the spatial and temporal heterogeneity of the TME, which can weaken or distort local gradients and reduce targeting reproducibility. Therefore, future autonomous targeting systems will likely require the integration of multiple targeting cues or combination with external guidance and secondary recognition mechanisms to improve in vivo specificity and translational robustness.

#### Collective and swarm navigation

While individual robots exhibit remarkable active delivery capabilities, they face inherent limitations in motion, payload, and imaging contrast [128–130]. Inspired by collective behaviors in nature, such as ant colonies and bird flocks, researchers have proposed “swarm cooperative navigation”, a paradigm where numerous robots form a functional swarm to accomplish complex tasks unattainable by a single agent [131–133]. This strategy is not merely a numerical aggregation; rather, it enables the emergence of novel functions and behavioral patterns through interagent interactions and coordinated responses.

Formation and Reconfiguration: The initial step in swarm behavior is formation. Under external driving fields (e.g., a rotating magnetic field), individual robots self-assemble into stable, ordered patterns like chains and vortices via magnetic dipole–dipole and hydrodynamic interactions [134,135]. Critically, these swarm morphologies are reconfigurable. By precisely modulating external field parameters (frequency, direction, and intensity), the swarm can be actively controlled to elongate, contract, split, or merge, enabling amoeba-like locomotion to navigate complex environments. For automated control, advanced feedback systems, such as fuzzy logic controllers, have been implemented. These controllers automatically adjust field parameters based on the real-time error between the observed and desired swarm shape, achieving fast, stable, closed-loop control of the swarm’s configuration [136].

Swarm Locomotion Control: Once a stable and adaptable formation is achieved, the next objective is to guide the collective entity toward its target. A stable swarm is typically controlled as a single entity for locomotion [137]. Control algorithms often rely on real-time tracking of the swarm’s center of mass, using a closed-loop controller to guide it along a predetermined trajectory [138]. This approach, which treats the swarm as a single rigid body, simplifies the control problem and has been demonstrated in applications such as intravascular targeted drug delivery and thrombus removal [139].

Independent and Autonomous Navigation: Progressing beyond unified rigid-body motion, the most advanced cooperative navigation paradigms demand higher autonomy for complex in vivo tasks. This more advanced cooperative navigation involves the ability to independently control multiple swarms or for the swarm itself to possess autonomous navigation capabilities. (a) By incorporating robots with different functionalities, heterogeneous swarms can be constructed to perform complex, multistep tasks. For example, a swarm can comprise “sensor-bots” for detecting environmental signals and “carrier-bots” for drug release. Upon detection of a pathological signal (e.g., low pH) by the sensor-bots, the carrier-bots are triggered to release their payload, achieving an integrated “sense-and-respond” smart delivery system [140]; (b) Collaborative task execution: Swarm behavior significantly enhances the physical capabilities and task efficacy of the robots [141]. For instance, a swarm of millions of magnetic microparticles can generate sufficient mechanical force to physically disrupt and remove a blood clot [139]. In cancer therapy, a swarm can more effectively penetrate the dense stromal matrix of a tumor through collective motion, enabling more uniform drug distribution into the tumor core; (c) Information exchange and autonomous decision-making: More advanced swarm intelligence involves information exchange between individual agents. Inspired by the “quorum sensing” mechanism in bacteria, researchers are exploring methods for inter-robot communication. This would enable swarms to make collective decisions—such as when to aggregate, disperse, or release a drug—based on local density and environmental feedback [142,143].

Despite these conceptual advantages, the clinical translation of swarm robotics faces significant biomedical and regulatory hurdles. First, tracking individual microrobots in vivo remains highly challenging due to the spatial resolution limits of current clinical imaging modalities; consequently, navigation typically relies on monitoring the swarm’s macroscopic center of mass or density, which inherently restricts the precise feedback control of individual agents. Second, the uneven distribution and attenuation of external power sources in deep tissues can lead to coordination failure, causing individual robots to detach from the swarm or fail to synchronize. Furthermore, multiagent systems introduce unique safety risks, including unpredictable abnormal aggregation, the potential for microvascular embolization in complex hemodynamic environments, and the risk of acute immune responses triggered by a high local concentration of carrier materials. Finally, the regulatory implications of multiagent systems present a substantial barrier. The nondeterministic, “emergent” behaviors inherent to advanced swarm intelligence contrast sharply with the strict, deterministic-outcome-based validation standards required by current medical device regulatory frameworks, necessitating the development of novel evaluation paradigms prior to clinical adoption.

## On-Demand Release Control

**Fig. 4.**
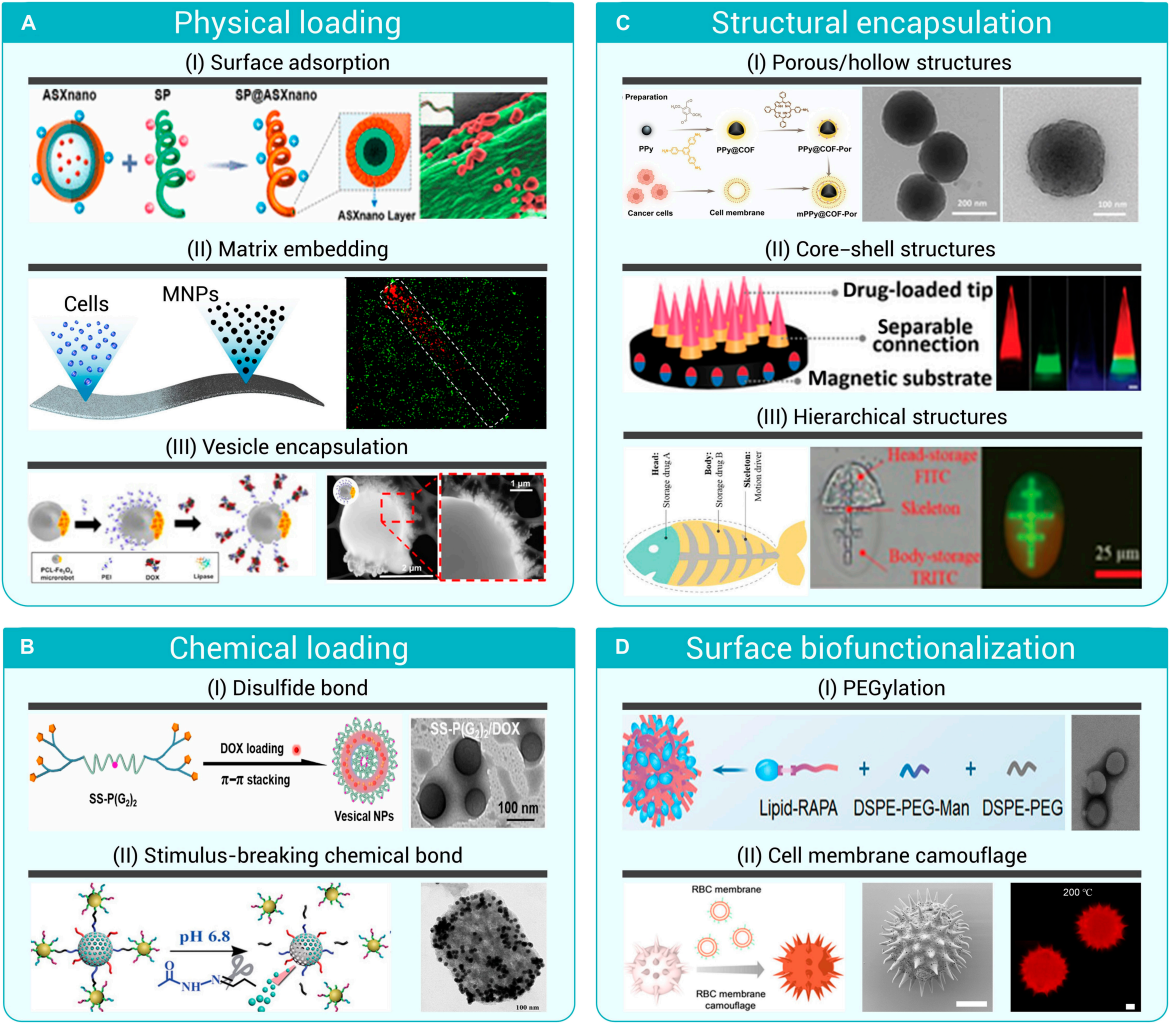
Drug loading and encapsulation. (A) Drug loading physical mechanisms. (I) Surface adsorption of the drug onto the microrobot. Scale bar: 500 nm. Reproduced or adapted with permission from Ref. [145], Copyright 2023 ACS. (II) Drug embedding within the microrobot’s body. Scale bar: 500 μm. Reproduced or adapted with permission from Ref. [45], Copyright 2025 IEEE. (III) Drug embedding within an attached vesicle. Scale bar: 2 μm (left) and 1 μm (right). Reproduced or adapted with permission from Ref. [149], Copyright 2022 Elsevier. (B) Drug loading chemical mechanisms. (I) Microrobots with disulfide bonds for redox-triggered drug release. Scale bar: 100 nm. Reproduced or adapted with permission from Ref. [151], (CC BY 4.0). (II) Microrobots with hydrazone bonds for pH-triggered drug release. Scale bar: 100 nm. Reproduced or adapted with permission from Ref. [154], Copyright 2023 Wiley. (C) Drug encapsulation strategies. (I) Drug is fixed in a hollow structure. Scale bar: 200 nm (left) and 100 nm (right). Reproduced or adapted with permission from Ref. [161], Copyright 2023 Wiley. (II) Drug is fixed in a core–shell structure. Scale bar: 1 cm. Reproduced or adapted with permission from Ref. [165], Copyright 2022 ACS. (III) Compartmentalized encapsulation of different drugs in distinct areas. Scale bar: 25 μm. Reproduced or adapted with permission from Ref. [166], Copyright 2023 Wiley. (D) Surface biofunctionalization of microrobots. (I) Microrobots with polyethylene glycol (PEG) surface modification. Scale bar: 200 nm. Reproduced or adapted with permission from Ref. [169], Copyright 2025 ACS. (II) Microrobots coated with cell membranes. Scale bar: 10 μm (left) and 5 μm (right). Reproduced or adapted with permission from Ref. [171], (CC BY-NC 4.0).

For precision therapy, robots must release therapeutic payloads with temporal, spatial, and dosage control. This on-demand release capability is a core advantage that distinguishes robots from traditional, passive drug delivery systems. By integrating advanced drug encapsulation technologies, stimuli-responsive mechanisms, and programmable strategies, robots are poised to elevate the precision of drug delivery to an unprecedented level (Fig. 4).

### Drug encapsulation and carrier design

The drug encapsulation system serves as the foundation for on-demand release. Its design must not only ensure the stability and biocompatibility of the drug during transport but also provide a controllable structural basis for subsequent precise release.

#### Drug loading strategies

##### Physical adsorption and entrapment

Physical loading strategies utilize noncovalent interactions—such as electrostatic attraction, hydrophobic interactions, or spatial confinement—to load therapeutic molecules onto or into robots [14,144]. The primary advantage of these methods is their mild processing conditions, which preserve the biological activity and structural integrity of macromolecular drugs like proteins and nucleic acids. Additionally, these approaches offer broad applicability across various drugs and carrier materials.

Surface Adsorption: This is the most direct physical loading method, primarily relying on physical forces such as electrostatic attraction, hydrophobic interactions, or hydrogen bonding between the drug molecules and the robot’s surface. For instance, researchers have loaded astaxanthin onto a *Spirulina*-based nano integrated system via electrostatic adsorption [145]. Others have adsorbed calcein-loaded liposomes onto the surface of helical artificial bacterial flagella through electrostatic interactions [146]. While the advantages of surface adsorption include straightforward operation and mild reaction conditions, it offers a relatively limited drug-loading capacity, and the drug is directly exposed to the external environment, making it susceptible to premature leakage caused by physiological fluid shear or pH variations.

Matrix Embedding: A more stable and protective physical loading method is to entrap drug molecules within the robot’s 3D matrix. Typically, researchers encapsulate therapeutic agents within robots fabricated from various types of hydrogels [45,147,148].

Vesicle Encapsulation: This method involves encapsulating drugs within carriers such as liposomes or polymer vesicles to form discrete, nanoscale drug-delivery units, which are then attached to the surface of robots. As biomimetic materials, liposomes exhibit excellent biocompatibility. Their bilayer membrane structure enables the encapsulation of hydrophilic (water-soluble) drugs within the aqueous core, as well as the loading of lipophilic (lipid-soluble) drugs within the membrane itself [149]. For instance, researchers have developed macrophage-based robots in which bioengineered bacterial outer membrane vesicles containing anticancer peptides are loaded into the macrophages [148].

##### Chemical conjugation and bonding

By forming stable and controllably cleavable covalent bonds, drug molecules can be precisely attached to the surface or framework of robots. The core of this method lies in the use of “linkers”—bifunctional molecules that react with both the robotic carrier and the drug molecule [150].

The most frequently used conjugation chemistries include amide bonds, ester bonds, and disulfide bonds. Among these, disulfide bonds are widely used to construct “reduction-sensitive” release systems because they can be selectively cleaved in the high glutathione (GSH) environment within tumor cells [151,152]. Additionally, researchers have designed breakable chemical bonds that respond to external stimuli. For example, photocleavable linkers can be broken by light of a specific wavelength to achieve light-controlled release [153]. Acid-sensitive hydrazone bonds can dissociate in the acidic TME and are used for tumor-specific drug delivery [154]. Other examples include redox-sensitive selenium bonds, reactive oxygen species (ROS)-cleavable thioether bonds, and enzyme-sensitive peptide bonds [155]. By rationally combining these conjugation strategies, robots can respond to different pathological signals, thereby enhancing the personalization and adaptability of the therapy.

##### Loading capacity and stability

Quantitative comparison of drug loading across microrobot platforms remains challenging because reported metrics are not always defined on the same basis. Nevertheless, available data indicate substantial variation depending on carrier architecture. For instance, a biohybrid algal microrobot carrying doxorubicin (DOX)-loaded PLGA nanoparticles showed a loading yield of 6.2 wt% and an encapsulation efficiency of 24.8% [66], whereas erythrocyte-based microswimmers achieved up to 78% DOX encapsulation efficiency [73], and a porous ZIF-8-based magnetic microrobot reported 93.9% ± 2.65% encapsulation efficiency [156]. An imaging-guided bioresorbable acoustic hydrogel microrobot also reached 62.0% of the theoretical 5-fluorouracil loading capacity [147]. Overall, these examples suggest that structural encapsulation generally provides higher loading efficiency than simple surface adsorption, although standardized reporting is still needed for direct comparison.

Stability under physiologically relevant conditions is also inconsistently reported. Liposome-functionalized artificial bacterial flagella showed no obvious fluorescence loss during active motion [157], whereas gelatin-based microrobots in simulated body fluid exhibited 22.96% release within 5 min and 32.93% within 10 min, followed by sustained release [158]. However, systematic evaluation under defined shear stress, serum exposure, and enzymatic degradation conditions remains limited. This is important because premature leakage under physiological shear, pH fluctuation, or enzymatic attack may substantially reduce the effective dose delivered to the target site.

From a translational perspective, premature leakage should be considered together with pharmacokinetics rather than as an isolated formulation issue. Although some active microrobot systems have shown improved tissue accumulation and prolonged local retention [66,147], systematic pharmacokinetic and biodistribution studies remain scarce [12,159]. Future work should therefore report not only loading efficiency and triggered release profiles but also circulation stability, off-target leakage, retention time, and in vivo biodistribution under realistic physiological conditions.

#### Architectural design of carriers

##### Structural encapsulation

By building specific micro- or nanostructures, it is possible to provide a physical “safe harbor” for drugs while simultaneously modulating their release behavior.

Porous/Hollow Structures: This represents the most direct method for increasing drug-loading capacity. Natural porous structures (such as the mesoporous shell of diatoms) or synthetically engineered porous materials (like metal–organic frameworks) are widely utilized. For instance, researchers have developed metal–organic frameworks-based robots that leverage their inherently high porosity to encapsulate large quantities of chemotherapeutic drugs [160,161]. Additionally, the internal cavity of tubular “microrockets”, fabricated via rolled-up nanotechnology or template electrodeposition, serves not only as a reaction chamber for generating propulsive bubbles but also as an ideal space for drug storage [162].

Core–Shell Structures: The core–shell architecture provides an additional protective layer for the drug payload [163,164]. For example, a drug-loaded core can be encapsulated within a pH-sensitive polymer shell, which dissolves to release the drug only when the robot reaches the acidic tumor environment [165]. Researchers have designed microneedle-shaped robots where the tip can be loaded with drugs while the main body provides propulsion and navigation, forming a functionally compartmentalized, core–shell-like structure [165].

Hierarchical/Multicompartment Structures: To achieve the synergistic or programmed release of multiple drugs, researchers are designing complex, multimodule integrated robots capable of carrying different therapeutic agents [166,167]. Recent advancements in manufacturing technologies have enabled the direct fabrication of robots with multiple independent chambers, offering unprecedented design freedom for complex combination therapy regimens [42,168].

##### Surface biofunctionalization

To help robots avoid immune clearance, extend circulation time, and improve targeting efficiency, researchers developed surface biofunctionalization techniques inspired by biological “camouflage”, effectively creating an “invisibility cloak” [10,11].

A classic immune evasion strategy is PEGylation, which involves modifying the robot’s surface with a layer of hydrophilic polyethylene glycol (PEG) polymer chains. This PEG layer forms a hydrophilic “protective shield” on the robot’s surface, which, through steric hindrance and its hydrophilic nature, effectively prevents the adsorption of plasma proteins (a process known as opsonization) and thus reduces the chances of being recognized and cleared by immune cells [169]. However, long-term application may induce the production of anti-PEG antibodies, leading to the “accelerated blood clearance effect”, which limits its long-term clinical use [170].

Current research is heavily focused on cell membrane camouflage technology. This technique involves coating the surface of robots with natural cell membranes stripped from specific cells, such as RBCs, platelets, or even cancer cells. This method is exceptionally clever because it not only preserves all the biological markers on the cell membrane surface, such as proteins and carbohydrates, but also maintains the membrane’s natural topology. For instance, robots coated with RBC membranes can effectively evade phagocytosis by macrophages due to the presence of the “don’t eat me” signal protein (e.g., CD47) on their surface, achieving long-term in vivo circulation [171]. Platelet membranes can help the robots target sites of inflammation or the TME [172]. Furthermore, using cancer cell membranes for coating endows the robots with a unique “homotypic targeting” ability, allowing them to act like a “Trojan horse” to specifically recognize and bind to tumor cells of the same origin [173].

### Stimuli-responsive release mechanisms

**Fig. 5.**
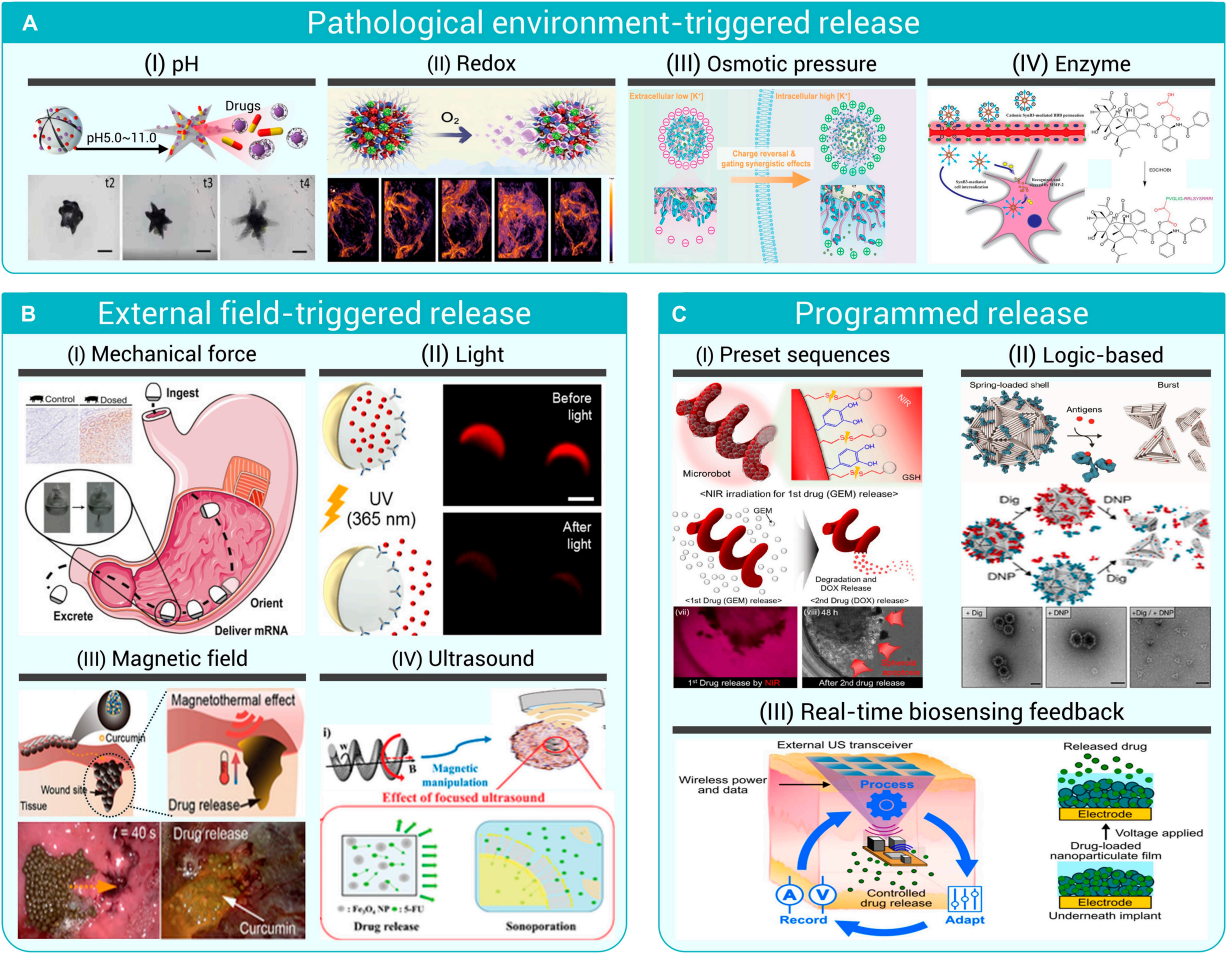
Controlled drug release. (A) Endogenous pathological environment-triggered drug release mechanisms. (I) pH-responsive drug release. Scale bar: 250 μm. Reproduced or adapted with permission from Ref. [176], (CC BY 4.0). (II) Redox-responsive drug release. Reproduced or adapted with permission from Ref. [184], Copyright 2024 Wiley. (III) Ion-responsive drug release. Reproduced or adapted with permission from Ref. [186], Copyright 2025 ACS. (IV) Enzyme-responsive drug release. Reproduced or adapted with permission from Ref. [179], (CC BY 4.0). (B) External field-triggered release mechanisms. (I) Drug release triggered by mechanical force. Reproduced or adapted with permission from Ref. [188], Copyright 2020 AAAS. (II) Light-triggered drug release. Scale bar: 500 μm. Reproduced or adapted with permission from Ref. [190], Copyright 2020 AAAS. (III) Drug release triggered by a magnetic field. Scale bar: 5 mm. Reproduced or adapted with permission from Ref. [191], Copyright 2024 Wiley. (IV) Ultrasound-triggered drug release. Reproduced or adapted with permission from Ref. [193], Copyright 2020 Wiley. (C) Programmed and controlled drug release strategies. (I) Sequential release of multiple drugs. Scale bar: 50 μm. Reproduced or adapted with permission from Ref. [195], Copyright 2023 ACS. (II) Logic-gated control of multidrug release. Scale bar: 100 nm. Reproduced or adapted with permission from Ref. [197], Copyright 2021 ACS. (III) Closed-loop controlled drug release. Reproduced or adapted with permission from Ref. [206], Copyright 2025 IEEE. US, ultrasound.

The essence of on-demand release control lies in the “stimulus-response” mechanism, whereby robots perceive specific internal or external signals and, in response, execute the command to release their therapeutic payload. This intelligent release behavior significantly enhances therapeutic precision and safety, while minimizing damage to healthy tissues (Fig. 5 and Table 2).

**Table 2.** Comparison of on-demand release capabilities

Release paradigm	Triggering mechanism	Implementation strategy and carrier design	Core advantages	Key challenges and limitations
Pathological environment-triggered	• Pathophysiological signals	• Stimuli-responsive materials	• High specificity and spontaneity • Autonomous operation	• Environmental heterogeneity • Risk of premature leakage • Limited control
External field-triggered	• External physical energy	• Energy-transducing materials • Energy-sensitive structures	• Spatiotemporally controllable • Dose controllable	• Limited penetration depth • Potential for tissue damage • Equipment dependency
Programmed release	• Preset sequences and logic • Real-time biosensing feedback	• Sequential release • Logic-gated release • Closed-loop feedback	• Complex combination therapies • Exceptional specificity • Personalized and adaptive dosing	• Fabrication complexity • Sensor reliability • System robustness

#### Endogenous triggers

Endogenous-triggered release strategies exploit the significant physicochemical differences between pathological and normal tissues. These differences are utilized as natural, built-in “release switches” to achieve spontaneous and highly selective drug release within the lesion area [174].

pH-Responsive Release: The anaerobic glycolysis in tumor tissues leads to an accumulation of lactic acid, creating a pH level that starkly contrasts with the physiological pH of normal tissues [113]. Researchers leverage this characteristic by designing robots that incorporate acid-sensitive chemical bonds (e.g., hydrazones, acetals) or are constructed from pH-sensitive polymers (e.g., chitosan and acrylic acid) [154,175]. When these robots reach the acidic TME, the chemical bonds break, or the polymers undergo conformational changes (such as swelling or dissolution), thereby triggering drug release [176].

Enzyme-Responsive Release: Many tumors overexpress specific enzymes, such as matrix metalloproteinases (MMPs), cathepsins, and phospholipases [177,178]. By linking the drug to a peptide or polymer substrate that can be cleaved by these specific enzymes, an enzyme-triggered drug release can be achieved. For example, a drug carrier “locked” by an MMP-2-sensitive peptide sequence will only be “unlocked” to release its payload upon reaching a tumor region with high MMP-2 expression [179].

Redox-Responsive Release: The concentration of GSH inside tumor cells (2 to 10 mM) is up to 1,000 times higher than in the extracellular environment (2 to 10 μM) [180]. Additionally, the levels of ROS, such as H₂O₂, are also significantly elevated in the tumor site [181]. This disparity is exploited by linking drugs to a carrier via disulfide, diselenide, or thioether/selenoether bonds. The resulting robot or micelle remains stable in the extracellular environment, which has low levels of GSH and ROS. Once it enters a tumor cell or a deep hypoxic region, the high GSH concentration rapidly cleaves the disulfide/diselenide bonds [182], or high ROS levels oxidize and break the thioether/selenoether bonds [183]. This triggers the rapid release of the active form of the drug within the cytoplasm, achieving tumor-specific enhanced delivery and therapy. Based on this principle, dual-responsive polymeric micelles that react to both hypoxia and ROS can achieve enhanced drug release and therapeutic effects in the deep regions of tumors [184].

Osmotic Pressure-Responsive Release: Pathological tissues often exhibit abnormal ion homeostasis or osmotic environments, such as elevated Ca^2+^ levels in inflamed areas [185], accumulation of extracellular K^+^ in tumors [186], and disruption of extracellular ion gradients following cerebral ischemia/injury [187]. These features provide “internal environment triggers” for robots, enabling the spontaneous release of drugs within specific tissues.

Although cues such as pH and ROS are attractive because they exploit pathological microenvironmental features, their specificity remains inherently limited in clinical applications. Acidification and elevated ROS levels are also associated with inflammation and ischemia-related diseases, rather than being unique to tumors. Accordingly, these endogenous cues are better considered context-enriching signals that may enhance local selectivity, rather than absolutely tumor-specific triggers for drug release [127,128].

#### Exogenous triggers

External physical fields like light, magnetic fields, and ultrasound provide a direct, active, and precise means of controlling drug release. An operator can precisely control the application time, location, and intensity of the physical field from outside the body, enabling “remote control” of drug release with high spatiotemporal resolution and noninvasiveness.

Mechanically Triggered Release: This approach utilizes the robot’s motility to trigger drug release through direct physical interaction. For instance, robots equipped with microneedles or sharp structures can be driven by external fields (e.g., magnetic fields) to physically penetrate tissue barriers or even cell membranes, directly “injecting” the drug at the target site [26,188].

Light-Triggered Release: Near-infrared (NIR) light, in particular, is an excellent external stimulus source due to its good tissue penetration, allowing for drug release control with high spatiotemporal resolution. By integrating photothermal conversion agents like gold nanoparticles into robots, NIR light irradiation can generate localized heat (the photothermal effect). This heat can be used to melt or trigger a phase change in thermosensitive drug carriers, such as hydrogels, leading to rapid drug release [189]. Additionally, by covalently attaching drugs to robots using photo-unstable chemical bonds, specific wavelengths of light (usually ultraviolet) can precisely cleave these bonds, achieving “light-controlled” drug release (photocleavable linkers) [190].

Magnetic Field-Triggered Release: Under an alternating magnetic field (AMF), superparamagnetic nanoparticles (e.g., Fe_3_O_4_) efficiently generate heat due to magnetic moment relaxation, a phenomenon known as magnetic hyperthermia. By integrating these nanoparticles into thermosensitive polymer-based robots, the local temperature can be precisely controlled by applying an external AMF, thereby triggering pulsed or sustained drug release [191]. For magnetoelectric robots, which are composites of magnetostrictive and piezoelectric materials, an AMF can induce a localized electric field (the magnetoelectric effect). This local electric field can be used to disrupt the electrostatic adsorption between the drug and the carrier, leading to drug release [192].

Ultrasound-Triggered Release: Focused ultrasound can generate localized high temperatures, intense mechanical vibrations, and cavitation bubbles at the target area. These effects can disrupt the structural integrity of drug carriers like liposomes, microcapsules, and even the porous structure of robots, thereby releasing the drug. Furthermore, the mechanical effects of ultrasound can temporarily increase the permeability of cell membranes, a phenomenon known as “sonoporation”, which accelerates the penetration and absorption of drugs into cells. This is particularly important in fields like gene delivery [193,194].

By contrast, exogenous triggers generally offer stronger control over release timing and location, but their clinical feasibility depends on penetration depth, safety, and regulatory practicality. Among them, ultrasound appears relatively more translatable because it combines deep tissue penetration with established safety assessment frameworks based on thermal and mechanical indices and recognized IEC standards. Light-triggered release provides high spatiotemporal precision but remains more limited by tissue penetration, particularly in the NIR-I range, making it more suitable for superficial or endoscopically accessible applications, although NIR-II partially improves this limitation [189]. Magnetic triggering is also attractive for deep-tissue actuation, but its translation will require careful consideration of magnetic resonance–environment compatibility, device testing, and labeling requirements [191]. Overall, ultrasound and magnetic strategies appear closer to clinical translation, whereas many optical and endogenous-triggered systems remain comparatively more experimental.

### Programmed and controlled release strategies

Transitioning from basic single-stimulus responsiveness, programmed drug release strategies represent an evolution toward more intelligent robot-based drug delivery. Such strategies aim to mimic complex biological signaling pathways, enabling preprogrammed control over the timing, logic, and dosage of drug release. This allows for the execution of more sophisticated therapeutic tasks, such as synergistic multidrug therapy or adaptive drug administration based on real-time physiological feedback.

#### Sequential and logic-gated release

In the clinical practice of treating complex diseases such as cancer, single-drug therapies often have limited efficacy. In contrast, combination therapy can produce a synergistic therapeutic effect, where “1+1 > 2”, by targeting different signaling pathways and overcoming drug resistance. However, the optimal timing and sequence for different drugs may vary. Sequential and logic-gated release strategies are designed to meet the demands of such complex synergistic treatments. Their goal is to enable robots to act like “micro-pharmacists”, precisely regulating the release of multiple drugs according to a preprogrammed schedule. These programmable rules are primarily implemented through 2 distinct mechanisms.

1. Sequential Release: Through sophisticated structural design or by leveraging the differential response times of various materials, the sequential release of multiple drugs can be achieved.

Multi-Compartment/Multi-Layer Structures: This involves designing robots with multiple independent physical compartments, each loaded with a different drug and controlled by a “gate” with distinct properties [195]. For example, the gate of one compartment might be pH-sensitive, while another is responsive to NIR light. By applying different stimuli in sequence, the sequential release of the drugs can be realized.

Differential Degradation: By utilizing the varying degradation rates of different polymer materials in vivo, a multilayered drug-loading structure can be constructed. The outer layer, made of a rapidly degrading material, is loaded with the drug intended for initial release, while the inner layer, composed of a slowly degrading material, carries the subsequent drug. This naturally creates a temporal sequence for release [196].

2. Logic-Gated Release: Logic-gated release requires a robot to execute its release operation (the logical output) only after 1 or more specific conditions (the logical inputs) are met, analogous to a “logic gate” in an electronic circuit.

“AND-Gate” Logic: This mechanism demands the simultaneous presence of 2 or more stimuli to trigger drug release, significantly enhancing the specificity of the release [197]. For example, a robot could be engineered to release its payload only when it concurrently detects the low pH of the TME AND receives an externally applied NIR light irradiation. This dual-safeguard mechanism can drastically reduce off-target release in nontarget areas [197].

Molecular Communication and Swarm Logic: Within a robot swarm, more complex logical control can be achieved through chemical signaling. For instance, a “messenger” robot swarm, upon identifying a target, could release a specific chemical signal (Input A). Only when an “executor” robot swarm receives this signal AND has itself arrived at the target area (Input B) will it execute the drug release (Output C). This type of swarm behavior, based on molecular communication, lays the foundation for implementing distributed, decentralized intelligent therapeutic strategies [198].

#### Closed-loop feedback-controlled release

Ultimately, moving beyond the static rules of sequential and logic-gated systems, the pinnacle of programmed release is dynamic autonomy. Closed-loop feedback control is the goal of “smart” drug delivery, aiming to create “theranostic” robots that can autonomously sense, process, and actuate. Unlike open-loop control, which relies on preset programs, a closed-loop system can dynamically adjust the therapeutic regimen based on real-time in vivo physiological and pathological information, thereby achieving true personalized precision medicine [199]. This strategy requires the robot to be not just a drug carrier, but an integrated microscale platform for both diagnostics and therapy [200–202].

A complete closed-loop feedback control robot system typically comprises 3 core modules:

Biosensor Module (Sensing Unit): This serves as the “eyes” of the system, responsible for the real-time monitoring of key biomarkers in vivo. By integrating probes sensitive to specific molecules (such as aptamers or antibodies) or sensors (like pH electrodes or glucose sensors) onto the robot, it can acquire real-time data on the lesion microenvironment (e.g., pH, oxygen concentration, and specific enzyme activity) or systemic physiological states (e.g., blood glucose levels) [114,203].

Signal Processing and Decision-Making Module (Processing Unit): This acts as the “brain” of the system, responsible for interpreting the signals from the sensing module and making a decision to release the drug based on a predefined therapeutic algorithm (for example, when blood glucose exceeds a certain threshold [204]) [205]. In current early-stage research, this “brain” may be located ex vivo, communicating wirelessly with the robots inside the body [206,207].

Drug Release Actuator Module (Actuating Unit): This functions as the “hands” of the system, responsible for executing the command to release the drug. This module is typically composed of the various stimuli-responsive materials and structures previously described. Upon receiving a command from the processing unit, the actuator is triggered to release the drug in a precise dosage [208].

Despite these highly promising conceptual frameworks, translating logic-gated and closed-loop systems into clinical reality is heavily constrained by engineering limitations. First, system integration at the micro/nanoscale is exceptionally difficult; miniaturizing highly sensitive biosensors and onboard processing units without compromising their functionality or the robot’s mobility remains a primary bottleneck. Second, the efficacy of current experimental closed-loop systems is frequently limited by feedback accuracy and response latency. In dynamic in vivo environments, biosensors are highly susceptible to biofouling and signal drift, leading to delayed or inaccurate feedback that can miss the optimal therapeutic window. Finally, these intelligent systems must meticulously account for severe failure modes. A false-positive signal from a degraded sensor, or a logic-gate malfunction, could trigger an uncontrolled burst release of highly potent drugs, leading to severe localized or systemic toxicity. Therefore, ensuring fail-safe mechanisms and long-term sensor stability are absolute prerequisites before these autonomous theranostic platforms can be deemed clinically viable.

## Preclinical Applications

**Fig. 6.**
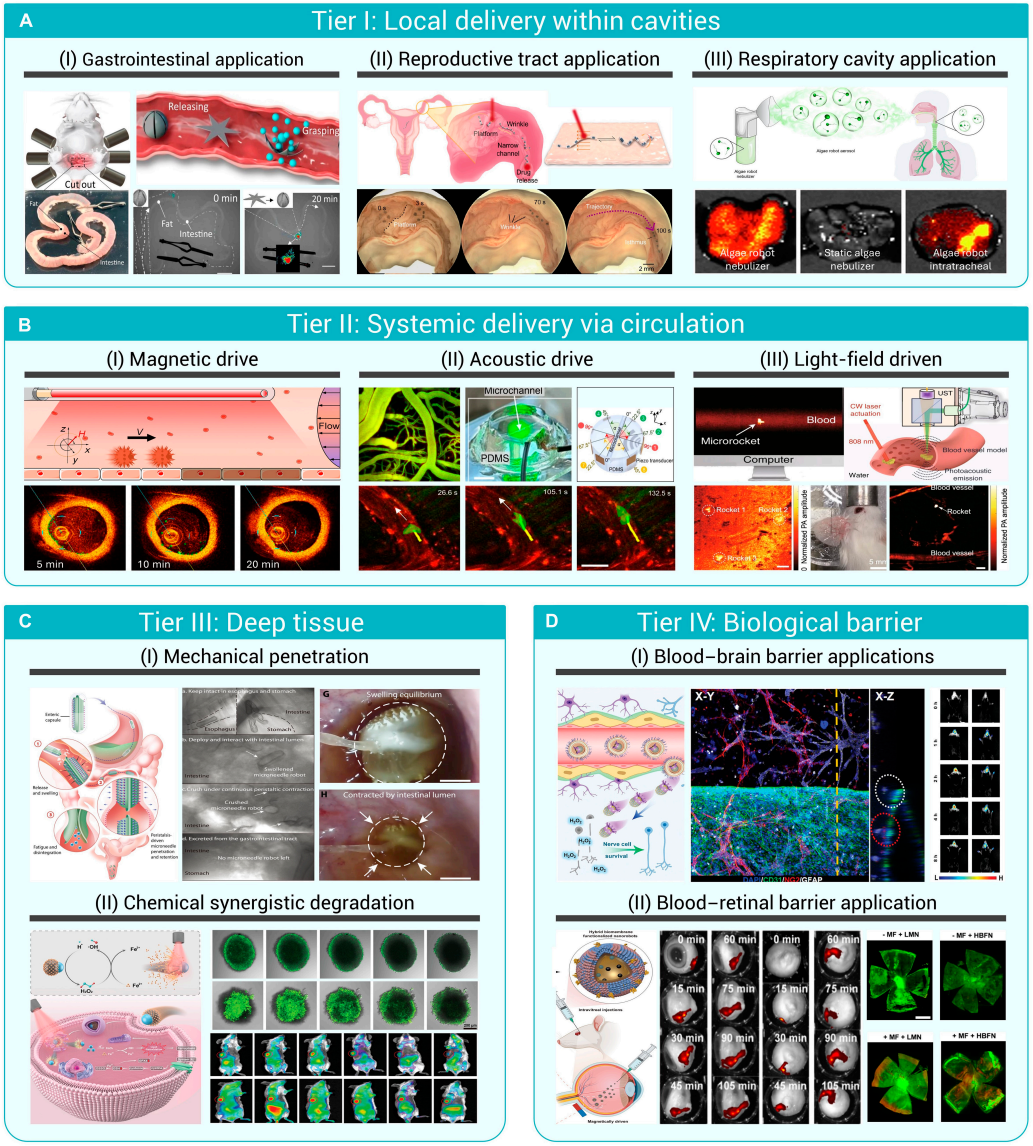
Applications of micro/nanorobots in drug delivery. (A) Local delivery within cavities. (I) Delivery into the intestinal tract. Scale bar: 2 cm. Reproduced or adapted with permission from Ref. [176], (CC BY 4.0). (II) Delivery into the reproductive tract. Scale bar: 2 mm. Reproduced or adapted with permission from Ref. [212], Copyright 2024 ACS. (III) Delivery into the lung. Scale bar: 5 mm. Reproduced or adapted with permission from Ref. [172], (CC BY 4.0). (B) Systemic delivery via circulation. (I) Delivery within jugular vessel. Scale bar: 1 mm. Reproduced or adapted with permission from Ref. [171], (CC BY-NC 4.0). (II) Delivery within the cerebral vessel. Scale bar: 50 μm. Reproduced or adapted with permission from Ref. [217], (CC BY 4.0). (III) Delivery within the superficial vessel. Scale bar: 100 μm. Reproduced or adapted with permission from Ref. [218], (CC BY 4.0). (C) Penetrate tissues. (I) Delivery by penetrating intestinal tissue. Scale bar: 5 mm. Reproduced or adapted with permission from Ref. [25], (CC BY 4.0). (II) Delivery by penetrating tumor tissue. Scale bar: 200 μm. Reproduced or adapted with permission from Ref. [224], Copyright 2024 Wiley. (D) Across biological barrier. (I) Delivery across the blood–brain barrier. Reproduced or adapted with permission from Ref. [228], Copyright 2024 Wiley. (II) Delivery across the blood–retinal barrier. Scale bar: 1 mm. Reproduced or adapted with permission from Ref. [233], Copyright 2025 ACS.

During in vivo delivery, robots must negotiate luminal clearance, immune recognition, dense ECMs, and selective barriers (e.g., blood–brain barrier and ocular and reproductive interfaces). Their ability to traverse these hurdles dictates distribution, retention, and efficacy; accordingly, in vivo challenges scale from local administration to systemic circulation, deep tissue penetration, and immune-privileged targets (Fig. 6).

### Tier I: Localized delivery within cavities

For in vivo applications, local delivery is the most fundamental and mature stage, enabling robots to achieve precise retention, targeted penetration, and in situ release within directly accessible cavities (gastrointestinal, reproductive, and pulmonary). This strategy establishes a high local drug concentration at lesions while reducing systemic exposure and toxicity, with controllable, minimally invasive operation and external-field guidance making it an optimal starting point for clinical translation [21]. This relative maturity is also reflected in stronger therapeutic readouts: For example, in an orthotopic mouse model of bladder cancer, radiolabeled urease-powered nanobots achieved an approximately 8-fold increase in tumor accumulation and produced about 90% tumor reduction after intravesical administration [118].

In the gastrointestinal tract—an established platform—magnetically guided capsules retain on gastric or intestinal mucosa and, carrying drug-delivery or electrochemical modules, deliver mRNA, proteins, or small molecules into the mucosal layer to enhance local concentration and limit off-target effects [188]. *Spirulina*-templated biohybrids use rotating magnetic fields for luminal adhesion and near-wall transport, releasing drug-loaded nanoparticles against infection or inflammation with high intracavitary retention and stability in acid, enzymes, and mucus; active locomotion and targeted adhesion further improve local gradients and efficiency [175,209].

In the urogenital tract, enclosed and partially fluid-filled cavities with regulated excretion favor reversible localized therapy: Urease-powered robots use urea-decomposition gas for self-propulsion to penetrate bladder epithelium and concentrate drugs at lesions [118]. Magnetically responsive flexible composites with hydrogel coatings reduce friction for safe, reversible bladder manipulation [210]. while sperm- or soft magnetic biohybrids provide strong propulsion and mucosal penetration to deliver hormones or antitumor drugs to the uterus and fallopian tubes in a minimally invasive, controllable manner [211,212].

In enclosed or low-flow spaces such as lungs and joints, robots offer flexible locomotion and precise control: Biohybrids achieve directional movement and drug accumulation in airways or alveoli to enhance local retention in animal models [172], and MnO₂-based systems in articular cavities modulate redox to reduce inflammation and promote repair. Flexible, biodegradable architectures ensure mechanical adaptability and in vivo compatibility for targeted interventions in constrained or quiescent regions [213]. These platforms typically adopt flexible, biodegradable architectures that ensure mechanical adaptability and in vivo compatibility, making them well suited for targeted interventions in anatomically constrained or physiologically quiescent spaces. Recent inhalation-based studies further support the translational promise of this tier: Nebulized biohybrid microrobots achieved stable lung distribution and prolonged retention exceeding 5 d in mouse lungs, and their therapeutic efficacy was subsequently demonstrated in a murine model of acute methicillin-resistant *Staphylococcus aureus* pneumonia [172].

### Tier II: Systemic delivery via circulation

Once robots enter systemic circulation, they face hemodynamic constraints and immune recognition; they must sustain propulsion and positioning under high shear while evading mononuclear phagocyte clearance, prompting a shift from single-field actuation to integrated systems that jointly optimize propulsion and immune evasion for precise, long-acting delivery [214]. Compared with localized delivery, this tier is translationally more demanding because improved navigation alone does not guarantee meaningful therapeutic benefit in the presence of rapid clearance, off-target deposition, and systemic exposure.

Magnetic fields enable remote orientation and trajectory control for aggregation and targeted accumulation in tumor-feeding vessels or inflamed sites [116], but protein corona formation shortens circulation time; cell membrane camouflage with erythrocyte, platelet [215], or neutrophil membranes, which preserve native receptors such as the CD47–SIRPα signal to reduce macrophage uptake and extend blood half-life, resulting in magnetically responsive and immune-stealth transport systems [70,171]. A representative translationally oriented example is human-scale magnetic microrobot navigation in the hepatic arteries of living pigs, where the distribution ratio in target liver lobes increased from 47.7% to 86.4% on the right side and from 52.2% to 84.1% on the left side, with a 1.7- to 2.6-fold increase in the number of microrobots reaching target lobes after multiple vascular bifurcations [216].

Acoustic propulsion supplies noncontact power in blood, driving robots via ultrasonic radiation force or streaming; focused ultrasound forms pressure nodes for directional aggregation, and magneto–acoustic hybrids combine magnetic guidance with acoustic thrust to maintain stable trajectories in branching, complex flows, achieving millimeter-scale focused intravascular delivery and higher local drug levels in vivo [79,217].

In superficial vessels or light-guide-accessible regions, optics serve as release and surface-chemistry switches: Integrating gold nanorods, phase-change units, or switchable bonds enables NIR-triggered heating/phase transitions for on-demand release and reversible “deshielding” from a stealth to a recognition state [218,219]. When combined with magnetic or acoustic navigation and surface PEGylation or membrane camouflage to suppress protein corona formation and complement activation, acid- or light-responsive deshielding achieves reversible activation with high site selectivity and cellular uptake [169,171].

### Tier III: Deep tissue penetration

The ECM of solid tissues, a 3D network of collagen, fibronectin, and proteoglycans, imposes substantial mechanical resistance, while elevated tumor interstitial fluid pressure drives outward flow that traps passively delivered drugs near vessels and limits access to deeper zones; thus, active locomotion with microenvironmental synergy is needed, requiring coordinated optimization of actuation, structure, and material interactions [220].

For deep penetration, robots use structural and material designs to actively traverse dense ECM. Helical magnetic robots under rotating fields mimic flagellar drilling through collagen and hyaluronic acid to extend penetration [221], microneedle-type robots perform puncture and release to create transient micropores and enhance interstitial diffusion [25], and flexible deformable robots based on gelatin methacryloyl, PEG diacrylate, or polycaprolactone–PEG adopt a squeeze-and-recover mode to minimize friction and damage [222]. When combined with magnetic or acoustic actuation, these soft-penetrating platforms integrate mechanical penetration with morphological adaptability for precise navigation and deep release across variable tissue densities. A representative example is a microorganism-powered microneedle micro-engine that increased drug delivery depth by more than 200%, reaching up to 1,000 μm below the skin, thereby illustrating the ability of active propulsion to enhance penetration beyond passive deposition [223].

Beyond mechanical means, robots can remodel ECM via matrix-degrading enzymes, an autocatalytic penetration strategy that lowers tissue resistance and improves diffusion in tumor organoids [118]. Enzyme-functionalized magnetic microrobots, such as alginate lyase systems, penetrate biofilm-like barriers and enhance local accessibility in vitro [12], and chemically responsive systems triggered by acid, ROS, or H₂O₂ soften or swell within lesions to increase deformability and permeability. Together, these mechanisms improve locomotion efficiency, drug distribution, and penetration gradients for treating deep-seated tumors [224].

### Tier IV: Targeting immune-privileged sites

In complex physiological systems, certain tissue regions—including the central nervous system, posterior eye, testes, and placenta—are enclosed by molecularly selective barriers that maintain immune homeostasis yet restrict drug delivery [225,226]. Therefore, achieving precise trans-barrier transport and localized therapy without disrupting local homeostasis is a transformative challenge in robot research. As a result, this tier currently remains the most translationally demanding and is still supported by comparatively limited therapeutic evidence.

The blood–brain barrier, formed by endothelial tight junctions, basement membrane, and astrocytic end-feet, excludes most exogenous molecules, with only a few small molecules passing passively [227]. Accordingly, brain delivery tasks focus on active transport or biomimetic migration: Magnetically actuated systems use gradient fields for vascular accumulation, surface “transcytosis-inducing ligands” enable receptor-mediated endocytosis [228], and immune-cell membrane camouflage supports chemotaxis-like, adhesion-mediated transendothelial migration [229]. In vivo models further show fluoroscopy-guided magnetic manipulation of blood-derived hydrogel fibers for personalized, trackable intracranial therapy [230], and soft, magnetically controllable microfibers function as implantable, movable interfaces with low foreign-body response and precise repositioning [231]. A notable step toward translation is the recent demonstration that magnetically driven biohybrid blood hydrogel fibers can be fluoroscopically tracked and used for DOX delivery to intracranial tumors in minipigs, providing one of the clearest examples of large-animal validation in an immune-privileged setting [230].

The posterior segment of the eye similarly presents a selective blood–retinal barrier that challenges nanoscale delivery [232]. To navigate micron-scale channels and viscoelastic matrices, flexible, deformable robots undergo controlled reconfiguration under magnetic or light actuation; photoresponsive platforms achieve 3D vitreous navigation and photothermal, subretinal release [233]. Coupled with optical coherence tomography and fluorescence feedback, these systems enable closed-loop positioning and dosage visualization, improving spatiotemporal precision while preserving barrier integrity and supporting applications in macular degeneration and retinal vascular disease [234]. Overall, these designs enhance the efficiency and spatiotemporal precision of local drug delivery while maintaining barrier integrity, showing significant potential for applications in treating conditions such as macular degeneration and retinal vascular diseases.

## Challenges and Bottlenecks to Clinical Translation

Despite significant progress, translating robots from animal experiments to human applications faces systemic barriers. These obstacles originate not only from limitations in materials, biocompatibility, and actuation control but are also deeply rooted in the complexities of the human immune system, ethical and regulatory oversight, and challenges in engineering reproducibility. In addition, because microrobot performance is often demonstrated under optimized experimental conditions, failure-prone scenarios such as propulsion loss, trajectory drift, insufficient imaging feedback, off-target accumulation, premature leakage, and biofouling-related functional deterioration remain insufficiently disclosed and discussed.

### Biosafety and biocompatibility

Translating robots to clinical applications requires overcoming biosafety and biocompatibility barriers. For long-term in vivo use, robots must coexist with blood, cells, and tissues without toxicity, immune rejection, or chronic inflammation. Despite advances in materials and biomimetic surfaces, validating long-term, system-level safety remains the principal bottleneck. In vivo, high ionic strength, complex enzymes, and redox gradients can trigger cellular stress or inflammatory cascades. Conventional magnetically actuated robots often use iron, cobalt, nickel, or their oxides (e.g., Fe_3_O_4_, CoFe_2_O_4_) [235]. While highly responsive, ion dissolution may cause mitochondrial damage and ROS accumulation, inducing apoptosis and localized inflammation [236]. Nanoscale magnetic particles can be retained by the reticuloendothelial system in the liver and spleen, creating risks of chronic toxicity and fibrosis, especially in chronic diseases or multidose regimens. To mitigate long-term risks and enhance clinical safety, research is shifting from inert metallic systems to biodegradable and absorbable platforms.

While advancing toward clinical use, immune compatibility and inflammation control are pivotal for long-term stability and safety. Immune-camouflaging strategies show promise but face challenges in stability, predictability, and regulatory feasibility within the dynamic human immune system. PEGylation prolongs circulation by forming a hydration barrier that inhibits plasma protein adsorption, yet repeated exposure can elicit anti-PEG antibodies and accelerated blood clearance [169]. Cell membrane coatings can achieve short-term immune evasion, but sustained exposure enables recognition of exogenous antigens [171], activating complement or chronic inflammation. Immune cell membranes such as neutrophil or monocyte membranes can add chemotaxis and lesion recognition with high specificity in immune-activated settings [71], although performance varies with individual immunity and pathology. A prevalent limitation is the absence of mechanisms for long-term compatibility and reversible clearance, since many efforts prioritize circulation time over metabolic clearance and immune reconstitution. Clinical translation is further hampered by the lack of systematic long-term biosafety assessments, as current work centers on short-term cytotoxicity or acute small-animal studies with limited data on multiorgan metabolism, chronic inflammation, and immune memory. Even for biodegradable systems with designed clearance, reports on actual long-term metabolic behavior and in vivo tracking remain scarce.

Beyond demonstrating that a robot is biodegradable, future studies need to define its long-term in vivo degradation kinetics in a quantitative and application-specific manner. The degradation profile should be evaluated together with the intended residence time, actuation mode, and delivery route, because overly rapid degradation can cause premature loss of propulsion, targeting accuracy, or cargo retention, whereas overly slow degradation can prolong tissue exposure and delay clearance [12,237]. Key parameters include mass loss, morphological evolution, decay of magnetic or mechanical performance, coupling between degradation and drug-release behavior, and the biodistribution, metabolism, and excretion of both matrix fragments and functional additives. Importantly, these processes should be tracked under physiologically relevant conditions and over sufficiently long observation windows, rather than being inferred only from short-term in vitro hydrolysis or endpoint histology. Such kinetic datasets will be essential for defining safe operating windows, predicting repeat-dose accumulation, and establishing clinically acceptable criteria for bioresorption and metabolic clearance.

### System robustness and controllability

In the translation of robots from proof of concept to clinical feasibility, system robustness and controllability are core bottlenecks to achieving safe and effective therapy. While the dual functions of motion and drug release have shown remarkable potential, their control precision and robustness in multiscale, dynamic, and highly heterogeneous biological environments still face severe challenges.

The in vivo navigation of robots confronts multiple challenges. First, the complex fluid dynamics [5,7,100,108]—such as the high-velocity, pulsatile, and non-Newtonian blood flow in vessels and the highly viscous interstitial fluid—render the robot’s motion dynamics highly nonlinear and difficult to model accurately. Second, micro/nanoscale stochastic disturbances [17,38,63,66,98], like Brownian motion, significantly impact the robot’s movement in low-Reynolds-number environments, thereby diminishing control precision. Furthermore, achieving real-time, high-precision localization and state estimation from medical imaging data with a low signal-to-noise ratio and poor contrast [12,14–16], especially in deep tissues, is exceedingly challenging and limits the effectiveness of closed-loop control. Lastly, traditional control algorithms exhibit insufficient adaptability in dynamic environments [99,100,105,111,112], struggling to cope with transient obstacles and physiological changes in vivo. This can cause the robot to deviate from its intended trajectory or even lose control. For robotic swarms, coordinating the interactions between individual agents (e.g., magnetic dipole forces and hydrodynamic coupling) within a high-dimensional parameter space, while simultaneously achieving collective motion, formation reconfiguration, and individual control under a single global input, remains a formidable challenge.

A core bottleneck for clinical translation is ensuring precise spatiotemporal control of on-demand drug release. This requires releasing a precise drug dose that matches the dynamic needs of the lesion’s heterogeneous microenvironment [4,12]. Concurrently, preventing premature drug leakage is a critical obstacle; the drug encapsulation system must maintain its structural integrity during long circulation times and under complex physiological conditions [5,6] (e.g., pH fluctuations, enzymatic activity, and shear forces) to avoid unintended leakage or aggregation due to nonspecific interactions. Moreover, the predictability of drug release kinetics remains a challenge. The high interindividual variability of the in vivo microenvironment makes it difficult to fully extrapolate results from in vitro models to the in vivo setting [1–3], leading to complexity and uncertainty in predicting release behavior.

### Ethical, regulatory, and standardization issues

As robotics advances in applications like drug delivery and oncology, a global consensus is emerging on the need to address ethical, regulatory, and standardization issues for clinical translation. The capacity of these technologies for autonomous movement, sensory perception, and intervention within the body introduces potential irreversible risks that pose unprecedented challenges to conventional regulatory frameworks for medical devices and pharmaceuticals [159,238].

On an ethical level, the evolution of robots from passive devices to “in-vivo intelligent agents” with decision-making capabilities raises fundamental questions regarding control and liability. Deviations from intended behavior or excessive payload release, potentially triggered by local environmental fluctuations, could lead to unforeseen harm. Consequently, a robust ethical framework must be established, emphasizing the principles of “controllable, reversible, and traceable” operation [159]. Furthermore, applications involving cell membrane camouflage or genetic manipulation must adhere strictly to established bioethical guidelines and informed consent protocols.

The absence of unified technical standards and data-sharing mechanisms severely hinders the reproducibility of research findings and increases the complexity of regulatory evaluation. Importantly, this problem is not limited to a lack of general reporting consistency but also reflects the absence of microrobot-specific benchmarking protocols. At present, no comprehensive standard exists for microrobot testing, and current evaluation still relies mainly on general translational frameworks such as Good Laboratory Practice procedures, International Organization for Standardization 10993 biocompatibility testing, and Food and Drug Administration pathways originally developed for conventional medical devices [238,239]. As a result, cross-study comparison remains difficult even for similar classes of robots, because core performance parameters such as actuation output, velocity, navigation accuracy, drug leakage, degradation rate, and in vivo half-life are often measured under nonequivalent experimental conditions [239,240]. Although task-specific benchmarks are beginning to emerge in areas such as imaging-based detection and tracking, the field still lacks a unified system-level benchmark that links laboratory performance with translational readiness.

## Future Perspectives

**Fig. 7.**
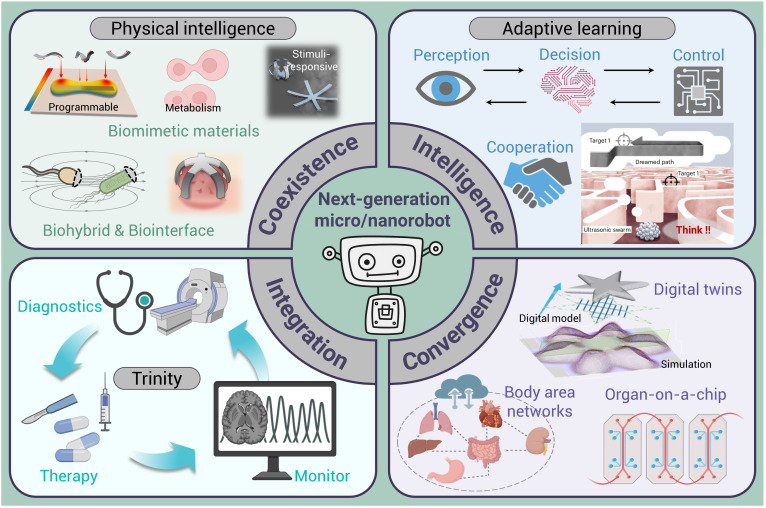
Prospects of medical micro/nanorobots. Reproduced or adapted with permission from Ref. [42], (CC BY NC). Reproduced or adapted with permission from Ref. [45], Copyright 2024 IEEE. Reproduced or adapted with permission from Refs. [59,111], (CC BY 4.0).

Autonomous robots for clinical applications are poised to evolve beyond their role as mere drug carriers into integrated, miniature targeted medical platforms capable of environmental sensing, intelligent decision-making, and multimodal therapy. Their future development necessitates simultaneous breakthroughs across multiple dimensions, including their physical embodiment, system intelligence, functional integration, and the pathway to clinical translation (Fig. 7).

### Enhancing environmental adaptability and biocompatibility

A fundamental paradigm shift in robot design is anticipated: a transition from an “invader” resisting the biological environment to a “symbiont” coexisting and coevolving with it. The core driver of this transformation is physical intelligence, which involves “encoding” sensing, decision-making, and response capabilities directly into the robot’s physical embodiment through sophisticated material design and structural engineering. This enables the robot to autonomously adapt and execute tasks through its physical interactions with the environment, obviating the need for complex on-board computational units.

The next generation of robots will focus on programmable, renewable, or biomimetic materials, such as multistimuli-responsive hydrogels, shape-memory polymers, and self-assembling nanocomposites. These materials can intelligently respond to physiological conditions—such as pH, temperature, redox potential, and specific biomolecules—to dynamically modulate their stiffness, surface chemistry, and drug-binding affinity. This capability allows the robots to exhibit organism-like adaptability, for instance, “softening” their bodies to pass through narrow blood vessels or “morphing” upon reaching the acidic TME to enhance tissue adhesion and penetration.

To achieve long-term, high-efficiency operation in vivo, the future direction of robot embodiment design will transcend the current paradigm of passive immune evasion, exemplified by PEGylation and static cell-membrane “camouflage”. It will advance toward a new paradigm: the construction of an adaptive biointerface capable of engaging in an active, dynamic “dialogue” with the host system. By being modified with materials that respond to immune signals and can adjust their surface protein conformations, or by leveraging synthetic biology to express specific immunomodulatory molecules, future robots will be able to actively manage their interactions with the immune system. This represents an evolution from “stealth” to “communication”, blurring the boundary between artificial devices and the biological environment and achieving a seamless integration with the living system.

### Advanced intelligence and autonomous control

At the individual level, the core evolution in the control of future robots will be a transformation from “teleoperated puppets” executing preprogrammed actions to intelligent agents capable of in vivo learning and adaptation. This involves tightly integrating sensing, computation, and action within the robot’s physical body, enabling it to learn and adapt through physical interaction with its environment rather than relying solely on preset programs. To realize this, developing artificial intelligence-integrated control architectures will be an indispensable framework. By embedding deep learning algorithms into closed-loop navigation systems, microrobots can autonomously process complex physiological feedback, moving beyond generic heuristic models to achieve true in situ adaptive decision-making and optimal trajectory planning in highly unpredictable environments [241–243]. Furthermore, by developing interactive feedback technologies, an intuitive communication channel can be established between the human operator and the robot. This will allow for high-level command input and real-time status feedback, merging human cognitive intelligence with the robot’s embodied intelligence to accomplish tasks in complex medical scenarios more efficiently and safely.

At the group level, intelligence will evolve from a centralized “command-and-execute” model to a decentralized “emergent behavior” paradigm. Future robot swarms will draw inspiration from the collaborative advantages of social insects and the immune system to build intelligent systems with distributed sensing, cooperative decision-making, and dynamic self-assembly capabilities. This will enable them to perform multistep, complex tasks in uncertain in vivo environments. The foundation of this paradigm is distributed decision-making: leveraging machine learning, especially deep RL, to empower each robot in the swarm to make local decisions based on local perceptions. This allows for complex collective behaviors—such as obstacle avoidance, formation maintenance, and task allocation—to emerge without central control. The prerequisite for achieving this distributed intelligence is the establishment of an effective in vivo communication network. Given the limitations of traditional radio frequency communication, individual robots may exchange information via chemical messengers, acoustic pulses, or local thermal signals to achieve advanced coordination and intelligent decision-making.

### Multifunctional integration and miniaturization

Future robots will transcend single functionalities, advancing toward multifunctional integration to create intelligent “diagnosis-therapy-monitoring” platforms that will enable more comprehensive and personalized medical services.

In situ noninvasive diagnostics: The integration of microbiosensors onto the robots will enable the real-time monitoring of key in vivo biomarkers (e.g., tumor markers, inflammatory factors, pH, and oxygen concentration), facilitating early disease diagnosis and precise pathological assessment.

Targeted Therapy: This involves integrating multimodal therapeutic capabilities, such as magnetic hyperthermia, photothermal therapy, gene therapy, and mechanical ablation, to achieve synergistic effects and enhance therapeutic outcomes. To support these multimodal therapies, the underlying propulsion mechanisms must also evolve toward hybrid actuation systems. Synergistically coupling multiple physical fields offers a robust framework to overcome the inherent penetration limits and environmental vulnerabilities of single-field actuation, ensuring reliable navigation and precise release in deep, heterogeneous tissues [244].

Therapeutic Monitoring: Robots will be capable of monitoring therapeutic effects in real time during treatment (e.g., changes in tumor volume, resolution of inflammation, and biomarker levels). This will enable the robots to autonomously adjust drug release dosages or patterns based on the therapeutic response, thereby realizing true personalized precision medicine.

### Interdisciplinary convergence and innovation

Future robots will benefit from deep integration with other cutting-edge technologies, constructing more complex and powerful intelligent healthcare systems.

Body Area Networks: By interconnecting robots, in vivo sensors, and wearable devices, a collaborative network can be formed to enable continuous physiological data monitoring, real-time communication, and distributed decision-making.

Digital Twins: Patient-specific virtual models (including organs, tissues, and the robotic system) will be constructed based on high-fidelity modeling and real-time data. These digital twins will be used to simulate and predict individualized therapeutic strategies.

Organ-on-a-Chip: Combining microfluidics with organ-on-a-chip technology will allow for the creation of highly biomimetic in vitro models. These platforms will be used for drug screening, toxicity assessment, and studying the behavior of robots, thereby accelerating preclinical validation and pharmaceutical research and development.

### Roadmap to clinical translation

The clinical translation of medical robots must follow a path that is gradual, risk-controlled, and guided by the establishment of standards.

Near-term: The initial focus should be on ex vivo applications or scenarios within easily accessible body cavities (e.g., the gastrointestinal tract, bladder, and eye), where validation can be performed under conditions that are visualizable, retrievable, and monitorable. These scenarios involve short intervention times and low local risks, which will facilitate the rapid accumulation of preclinical and early clinical data. This will also enable the exploration of precision diagnostics and therapeutics for high-value diseases such as digestive tract tumors and ophthalmic inflammation.

Mid- to Long-term: The strategy will expand to address more complex deep tissues and systemic diseases (e.g., cardiovascular, nervous system, and TME). This will necessitate the development of “micro-therapeutic units” capable of long-term operation within the blood or lymphatic systems to achieve dynamic navigation, in situ theranostics, and repeated on-demand release. Concurrently, it is crucial to establish comprehensive regulatory science and industry standards covering manufacturing consistency, biocompatibility, biodegradability, long-term safety, and ethical compliance. The simultaneous advancement of quality control and risk assessment frameworks will be essential to lay the foundation for commercial-scale production and widespread clinical adoption.

## Data Availability

The data are freely available upon request.
